# Symplectic Foliation Structures of Non-Equilibrium Thermodynamics as Dissipation Model: Application to Metriplectic Nonlinear Lindblad Quantum Master Equation

**DOI:** 10.3390/e24111626

**Published:** 2022-11-09

**Authors:** Frédéric Barbaresco

**Affiliations:** THALES Land & Air Systems, 19/21 Avenue Morane Saulnier, 78140 Vélizy-Villacoublay, France; frederic.barbaresco@thalesgroup.com

**Keywords:** Lie group thermodynamics, symplectic geometry, symplectic foliation, Poisson cohomology, Casimir function, transverse Poisson structure, entropy, heat equation, Koszul–Fisher metric, maximum entropy, symplectic geometry, exponential density family, transverse Poisson structure, Slodowy slices, Grothendieck–Brieskorn–Slodowy theorem, metriplectic model, Onsager–Casimir relations, affinity, thermodynamics fluxes and response coefficients, Clausius–Duhem inequality, Dirac dissipative brackets, Lindblad equation

## Abstract

The idea of a canonical ensemble from Gibbs has been extended by Jean-Marie Souriau for a symplectic manifold where a Lie group has a Hamiltonian action. A novel symplectic thermodynamics and information geometry known as “Lie group thermodynamics” then explains foliation structures of thermodynamics. We then infer a geometric structure for heat equation from this archetypal model, and we have discovered a pure geometric structure of entropy, which characterizes entropy in coadjoint representation as an invariant Casimir function. The coadjoint orbits form the level sets on the entropy. By using the KKS 2-form in the affine case via Souriau’s cocycle, the method also enables the Fisher metric from information geometry for Lie groups. The fact that transverse dynamics to these symplectic leaves is dissipative, whilst dynamics along these symplectic leaves characterize non-dissipative phenomenon, can be used to interpret this Lie group thermodynamics within the context of an open system out of thermodynamics equilibrium. In the following section, we will discuss the dissipative symplectic model of heat and information through the Poisson transverse structure to the symplectic leaf of coadjoint orbits, which is based on the metriplectic bracket, which guarantees conservation of energy and non-decrease of entropy. Baptiste Coquinot recently developed a new foundation theory for dissipative brackets by taking a broad perspective from non-equilibrium thermodynamics. He did this by first considering more natural variables for building the bracket used in metriplectic flow and then by presenting a methodical approach to the development of the theory. By deriving a generic dissipative bracket from fundamental thermodynamic first principles, Baptiste Coquinot demonstrates that brackets for the dissipative part are entirely natural, just as Poisson brackets for the non-dissipative part are canonical for Hamiltonian dynamics. We shall investigate how the theory of dissipative brackets introduced by Paul Dirac for limited Hamiltonian systems relates to transverse structure. We shall investigate an alternative method to the metriplectic method based on Michel Saint Germain’s PhD research on the transverse Poisson structure. We will examine an alternative method to the metriplectic method based on the transverse Poisson structure, which Michel Saint-Germain studied for his PhD and was motivated by the key works of Fokko du Cloux. In continuation of Saint-Germain’s works, Hervé Sabourin highlights the, for transverse Poisson structures, polynomial nature to nilpotent adjoint orbits and demonstrated that the Casimir functions of the transverse Poisson structure that result from restriction to the Lie–Poisson structure transverse slice are Casimir functions independent of the transverse Poisson structure. He also demonstrated that, on the transverse slice, two polynomial Poisson structures to the symplectic leaf appear that have Casimir functions. The dissipative equation introduced by Lindblad, from the Hamiltonian Liouville equation operating on the quantum density matrix, will be applied to illustrate these previous models. For the Lindblad operator, the dissipative component has been described as the relative entropy gradient and the maximum entropy principle by Öttinger. It has been observed then that the Lindblad equation is a linear approximation of the metriplectic equation.

## 1. Symplectic Statistical Mechanics Introduced by Jean-Marie Souriau

By considering Lie group coadjoint orbits as a homogeneous symplectic manifold, Jean-Marie Souriau [1,2,3,4,5,6,7,8,9,10,11,12] extended the traditional idea of a canonical ensemble given by Gibbs to the case of a symplectic manifold, defining a generalized Gibbs states parameterized by an element of the Lie algebra. A generalized information geometry by Souriau includes inequalities of convexity along with a number of other conventional thermodynamic features, such as temperature being a component of the Lie algebra and heat being a component of the dual space of the Lie algebra. In the case of non-commutative groups, specific characteristics emerge: specific cohomological relations appear in the Lie group algebra. A novel symplectic theory of information geometry and theory of heat is then given by the Souriau model deduced from the foliation. We introduce this new foliation theory of heat and information geometry using symplectic geometry and Poisson deduced from “Lie group thermodynamics”. For basic references to Souriau’s model, we invite you to read the author [13,14,15,16,17,18,19,20,21,22,23,24,25,26], C.M. Marle [27,28,29,30,31,32,33,34,35,36], G. de Saxcé [37,38,39] and more recent studies [40,41,42,43]; for symplectic geometry, J.L. Kosul [44] and P. Cartier [45]; and for an extension to quantum thermodynamics, [46,47,48,49,50,51]. In the fourth chapter on “Statistical Mechanics” of his book *Structure of Dynamical Systems* published in 1969, Souriau first introduced symplectic statistical mechanics. We will infer a geometric heat equation from this archetypal model, and we have discovered a pure geometric entropy definition, which manifests entropy as a Casimir function [52]. By using the KKS 2-form, introduced by Kirillov, Kostant and Souriau, in the affine situation via a symplectic cocycle, the method also enables generalizing the Fisher metric for Lie groups. The coadjoint orbits and the Souriau moment map are crucial components of this concept. Ontologically, this model offers the same geometric structures for the theory of probability, model of statistical mechanics and information geometry. Entropy gains a geometric foundation as a function indexed by the dual space of the Lie algebra through the moment map and in terms of foliations. For a Lie group G operating on a manifold having a symplectic form by symplectomorphisms, Souriau established the extended Gibbs rules. By extending the Fisher and Koszul metrics [53] from the theory of information geometry with the definite positiveness of the Souriau tensor, we will be able to explain the second principle of thermodynamics. Souriau entropy is invariant when a group is acting on a homogeneous symplectic manifold and when a coadjoint operator acting on heat has affine equivariance. These equations are ubiquitous and may be of significant relevance in mathematics, according to Souriau. Koszul–Poisson cohomology characterizes entropy as a Casimir function. The entropy level sets are formed by the coadjoint orbits as symplectic leaves that emerge from the dual space of the Lie algebra by KKS 2-form. The dynamics transverse to these symplectic leaves is dissipative, and dynamics remaining on symplectic leaves is non-dissipative and help us to understand this Lie group thermodynamics within the context of open system out-of-equilibrium thermodynamics.

For the symplectic leaves, the transverse Poisson structure, which is endowed with its Poisson structure, will be taken into consideration in the second half of our discussion on the dissipative symplectic theory of heat and information. There are three different types of dissipation: thermal diffusion with energy conservation and entropy production through heat transfer; viscosity, which takes energy from the system (e.g., Navier–Stokes equation); and transport equations with collision operators. Hamiltonian dynamics describes systems that maintain energy throughout the phase. Dissipative effects, which are irreversible changes from a thermodynamic perspective, cannot be accounted for in classical Hamiltonian systems (dissipative dynamics that do not preserve energy). The metriplectic bracket was first introduced in 1983 by A. N. Kaufman [54] and P. J. Morrison [55,56,57], providing both conservation of energy and entropy production, and it reduces to the traditional Poisson bracket formalism in the limit of no dissipation. Parallel axiomatization of this model has been performed by Grmela [58,59] and Öttinger (with a method called GENERIC). Entropy in these models is assumed to be a Casimir function, as in the Souriau model. These types of systems that follow both the first principle and the Clausius second principle of thermodynamics are included in metriplectic dynamics.

Baptiste Coquinot [60,61] recently developed a new foundation theory for dissipative brackets by taking a broad perspective from non-equilibrium thermodynamics. He accomplished this by first considering variables for building the bracket of the metriplectic flow and by developing a methodical approach for dissipative brackets. Based on non-equilibrium thermodynamic equations developed by Onsager and Casimir [62,63,64,65,66] for systems near thermal equilibrium for irreversible processes, Baptiste Coquinot developed metriplectic dissipative brackets. The Onsager–Casimir reciprocity relations describe time-reversal invariance at the microscopic scale for the macroscopic quantity relaxation close to thermodynamic equilibrium in the linear regime and link time reversibility at the microscopic scale with a symmetry property of corresponding evolution equations at the macroscopic scale. With the macroscopic evolution equation described by variational principle as a flow of gradient or equation of maximum entropy production, the Onsager–Casimir relations assume that, for the thermal fluctuations of macroscopic quantities, correlation functions decay with respect to the equations of macroscopic relaxation. Baptiste Coquinot’s concept was to elaborate with naturally thermodynamic variables that are preserved in place of the typical Hamiltonian variables. By a formal equivalence demonstration between the thermodynamics of out-of-equilibrium equilibrium and metriplectic dynamical systems, Baptiste Coquinot demonstrates that the pseudometric of the dissipative bracket is exactly equivalent to the second principle of thermodynamics and relations elaborated by Onsager and Casimir. By deriving a generic bracket for dissipation from the fundamental first principle of thermodynamics, Coquinot’s development demonstrates that brackets for dissipation are canonical for out-of-equilibrium equilibrium, just as Poisson brackets are canonical in the case of dynamics described by Hamilton. This general Coquinot dissipative bracket includes non-equilibrium thermodynamic theories, such as the one initially proposed for the metriplectic model. According to Baptiste Coquinot, entropy, a Casimir invariant, plays a similar role to the Hamiltonian in analytical mechanics. The pseudometric’s non-negativity assures that entropy continues to increase in accordance with the second principle of thermodynamics. According to Baptiste Coquinot, the natural variables that make up the phase space basis are different from one another from both a Hamiltonian and a thermodynamic standpoint, but one can modify the variables by using the identity of thermodynamics to obtain a bracket in any phase space variables. The first principle of thermodynamics is well formulated in the Coquinot bracket for dissipation, with all the characteristics (bilinearity, symmetry and degeneracy). Baptiste Coquinot was the first to notice these properties for a metriplectic dynamical system.

We shall use first Dirac’s dissipative bracket theory [67,68,69,70,71] for constrained Hamiltonian systems to refer to transverse structure. For Lagrangian systems with degenerate Lagrangians, Paul Dirac introduced the generalized Hamiltonian dynamics in 1950. In this case, we can endow the system phase space with two Poisson brackets: the Poisson bracket deduced from its symplectic structure, and the Dirac bracket. Cristel Chandre has more recently examined these Hamiltonian systems with constraints within the context of the theory developed by Paul Dirac, demonstrating that the identity introduced by Jacobi arises from requiring that the constraints be invariants of Casimir, in any case of invertibility of the Poisson bracket matrix between constraints. Cristel Chandre notes that this guarantees the Jacobi identity.

After Paul Dirac, Michel Saint-Germain [72,73] studied the transverse Poisson structure in his PhD work, which was inspired by Fokko du Cloux’s seminal works on the associative algebra structure [74,75,76,77,78,79,80,81,82,83]. This approach differs from the metriplectic approach. In continuation of the works of Saint-Germain, in 2005, Hervé Sabourin investigated the polynomial nature, transverse to nilpotent adjoint orbits, of the Poisson structures in the case of a complex semisimple Lie group with the introduction of a few nilpotent orbit families with transverse structures that are quadratic. P. A. Damianou previously proposed as early as 1989 that a polynomial property should characterize these previous transverse Poisson structures. H. Sabourin provided complements with polynomial transverse structures in 2005, making use of Lie algebras’ machinery for semisimple Lie groups. Independent Casimir functions associated with the transverse Poisson structure are constrained to the transverse slice of the Lie–Poisson structure [84]. It is based on a Slodowy–Brieskorn theorem that was extended by Sabourin from an Alexandre Grothendieck conjecture [85,86,87,88,89]. Hervé Sabourin [90,91,92,93] has demonstrated that the transverse Poisson and the determinantal structures, which are built using these previous Casimirs, are on the symplectic leaf transverse slice that have Casimir functions, two polynomial Poisson structures. Both structures have the same quasi-degree (up to a constant multiple). On the basis of a Slodowy theorem, he also demonstrated that the transverse Poisson is fundamentally given by a 3 × 3 matrix, with skew-symmetric property, that is closely connected to the polynomial that characterizes the singularity. He also focused on the subregular and minimum orbit cases.

To conclude, we shall explore the equation introduced by Lindblad, the Hamiltonian Liouville equation with an additional dissipative part, operating on the quantum density matrix, to illustrate earlier models. To demonstrate that the equation developed by Lindblad can be written as a damped Hamiltonian system and the GENERIC (General Equation for Non-Equilibrium Reversible and Irreversible Coupling) model, which was developed by Grmela and Öttinger, we will remind the reader of the study performed by Markus Mittnenzweig [94,95,96,97,98,99]. The Lindblad dissipative part operator has been represented as the relative entropy gradient, indicating that the maximum entropy principle can lead to equilibria. Classically dissipative quantum systems are described by the linear quantum master equation in Lindblad form, which is the most common. However, H.C. Öttinger [100,101] has pointed out that this equation’s essential flaw—invoking an inaccurate “quantum regression hypothesis”—has been known for around 30 years. This issue has been solved for a heat bath connected to a quantum system by the inclusion of a nonlinear master equation linked to a “modified quantum regression hypothesis” by H. Grabert [40,102]. The projection operator method has been used to obtain this modified master equation. The nonlinear master equation, obtained in this way, is not restricted to temperatures with high values where minor quantum effects inevitably occur. The quantum master equation can really be used down to arbitrary temperatures of low values if the connection due to friction to the heat bath weakens enough, as has been demonstrated. After formulating the nonlinear master equation that is thermodynamically consistent, one can search for unique circumstances where one can derive precise or approximate linear master equations. H. Grabert has conducted the same kind of studies and came to the conclusion that the well-known Lindblad form is not a good master equation.

## 2. List of Notations

We will use the following notation in the paper:

- ***Lie and dual Lie algebras:***

Lie algebra: $g=T_{e}G$

Dual space of Lie algebra $g^{*}$

- ***Coadjoint operator:***

$$\begin{array}{l} Ad_{g}^{*}={\left(Ad_{g^{-1}}\right)}^{*}\\ \mathrm{with}\;\langle Ad_{g}^{*}F,Y\rangle =\langle F,Ad_{g^{-1}}Y\rangle ,\forall g\in G,Y\in g,F\in g^{*} \end{array}$$

- ***Moment map:***

$$J(x):M\to g^{*}\;\mathrm{such}\;\mathrm{that}\;J_{X}(x)=\langle J(x),X\rangle ,\;X\in g$$

- ***Souriau 1-cocycle:***

$$\theta \left(g\right)$$

- ***Souriau 2-cocycle:***

$$\tilde{\Theta }\left(X,Y\right)=J_{\left[X,Y\right]}-\left\{J_{X},J_{Y}\right\}$$

where

$\begin{array}{l} g\times g\to ℜ\\ X,Y\mapsto \tilde{\Theta }\left(X,Y\right)=\langle \Theta (X),Y\rangle \;\mathrm{with}\;\Theta (X)=T_{e}\theta \left(X(e)\right) \end{array}$

- ***Affine coadjoint operator:***

$$Ad_{g}^{\#}(.)=Ad_{g}^{*}(.)+\theta \left(g\right)$$

- ***Poisson Bracket given by KKS 2-form***

$$\left\{F,G\right\}\left(X\right)=\langle X,\left[\frac{\partial F}{\partial X},\frac{\partial G}{\partial X}\right]\rangle$$

- ***Affine Poisson bracket:***

$${\left\{F,G\right\}}_{\tilde{\Theta }}\left(X\right)=\langle X,\left[\frac{\partial F}{\partial X},\frac{\partial G}{\partial X}\right]\rangle +\langle \Theta \left(\frac{\partial F}{\partial X}\right),\frac{\partial G}{\partial X}\rangle$$

## 3. Information Geometry Foundation of Souriau “Lie Group Thermodynamics”

For geometric statistical mechanics, we will discuss how to introduce statistical tools for Lie groups, and more specifically how to define the extension of Gauss density as the maximum entropy density of Gibbs. 

Amari has demonstrated that the information matrix of Fisher is the Riemannian metric for an exponential family as follows:

(1) $$g_{ij}=-{\left[\frac{\partial^{2}\Phi }{\partial \theta_{i}\partial \theta_{j}}\right]}_{ij}\;\mathrm{with}\;\Phi (\theta )=-\log\int_{\mathbb{R}}e^{-\langle \theta ,y\rangle }dy$$

The Legendre transform provides a dual potential as Shannon entropy:

(2) $$S(\eta )=\langle \theta ,\eta \rangle -\Phi (\theta )\;\mathrm{with}\;\eta_{i}=\frac{\partial \Phi (\theta )}{\partial \theta_{i}}\;\mathrm{and}\;\theta_{i}=\frac{\partial S(\eta )}{\partial \eta_{i}}$$

where $\Phi (\theta )=-\log\int_{R}e^{-\langle \theta ,y\rangle }dy=-\log\psi \left(\theta \right)$ is the classical function for cumulant generation.

Through an affinely invariant Hessian metric on a sharp convex cone, J.L. Koszul and E. Vinberg have introduced a generalization, though the concept of characteristic function:

(3) $$\begin{array}{l} \Phi_{\Omega }(\theta )=-\log\int_{\Omega^{*}}e^{-\langle \theta ,y\rangle }dy=-\log\psi_{\Omega }(\theta )\;\mathrm{with}\;\theta \in \Omega \;\mathrm{sharp}\;\mathrm{convex}\;\mathrm{cone}\\ \psi_{\Omega }(\theta )=\int_{\Omega^{*}}e^{-\langle \theta ,y\rangle }dy\;\mathrm{with}\;\mathrm{Koszul}-\mathrm{Vinberg}\;\mathrm{Characteristic}\;\mathrm{function} \end{array}$$

The name “characteristic function” has been named by Ernest Vinberg [103,104]:

(4) $$\begin{array}{l} \mathrm{Let}\;\Omega \;\mathrm{be}\;a\;\mathrm{cone}\;\mathrm{in}\;U\;\mathrm{and}\;\Omega^{*}\;\mathrm{its}\;\mathrm{dual},\;\mathrm{for}\;\mathrm{any}\;\lambda >0,H_{\lambda }(x)=\left\{y\in U/\langle x,y\rangle =\lambda \right\}\\ \mathrm{and}\;\mathrm{let}\;d^{\left(\lambda \right)}y\;\mathrm{denote}\;\mathrm{the}\;\mathrm{Lebesgue}\;\mathrm{measure}\;\mathrm{on}\;H_{\lambda }(x):\\ \psi_{\Omega }(x)=\int_{\Omega^{*}}e^{-\langle x,y\rangle }dy=\frac{\left(m-1\right)!}{\lambda^{m-1}}\int_{\Omega^{*}\cap H_{\lambda }(x)}d^{\left(\lambda \right)}y \end{array}$$

There exists a bijection $x\in \Omega \mapsto x^{*}\in \Omega^{*}$, satisfying the relation $\left(gx\right){\;}^{*}={\;}^{t}g^{-1}x^{*}$ for all $g\in G\left(\Omega \right)=\left\{g\in GL(U)/g\Omega =\Omega \right\}$ the linear automorphism group of Ω, and $x^{*}$ is:

(5) $$x^{*}=\int_{\Omega^{*}\cap H_{\lambda }(x)}yd^{\left(\lambda \right)}y/\int_{\Omega^{*}\cap H_{\lambda }(x)}d^{\left(\lambda \right)}y$$

We can observe that $x^{*}$ is the center of gravity of $\Omega^{*}\cap H_{\lambda }(x)$. We have the property that $\psi_{\Omega }(gx)={\left|\det(g)\right|}^{-1}\psi_{\Omega }(x)$ for all $x\in \Omega ,g\in G\left(\Omega \right)$ and then that $\psi_{\Omega }(x)dx$ is an invariant measure on Ω. Writing $\partial_{a}=\sum_{i=1}^{m}a^{i}\frac{\partial }{\partial x^{i}}$, one can write:

(6) $$\partial_{a}\Phi_{\Omega }(x)=\partial_{a}\left(-\log\psi_{\Omega }(x)\right)=\psi_{\Omega }{\left(x\right)}^{-1}\int_{\Omega^{*}}\langle a,y\rangle e^{-\langle x,y\rangle }dy=\langle a,x^{*}\rangle \;,\;a\in U,x\in \Omega$$

Then, the tangent space to the hypersurface $\left\{y\in U/\psi_{\Omega }(y)=\psi_{\Omega }(x)\right\}$ at x∈Ω is given by $\left\{y\in U/\langle x^{*},y\rangle =m\right\}$. For x∈Ω,a,b∈U, the bilinear form $\partial_{a}\partial_{b}\log\psi_{\Omega }(x)$ is symmetric and positive definite, so that it defines an invariant Riemannian metric on Ω.

Jean-Marie Souriau extended these relationships in geometric statistical mechanics to ensure the covariance of Gibbs density with regard to the action of the Lie group. The Souriau model preserves earlier structures:

(7) $$I(\beta )=-\frac{\partial^{2}\Phi }{\partial \beta^{2}}\;\mathrm{with}\;\Phi (\beta )=-\log\int_{M}e^{-\langle U(\xi ),\beta \rangle }d\lambda_{\omega }\;\mathrm{and}\;U:M\to g^{*}$$

We can observe that the Legendre transform is preserved:

(8) $$S(Q)=\langle Q,\beta \rangle -\Phi (\beta )\;\mathrm{with}\;Q=\frac{\partial \Phi (\beta )}{\partial \beta }\in g^{*}\;\mathrm{and}\;\beta =\frac{\partial S(Q)}{\partial Q}\in g$$

It should be noted that the definition of entropy is “Legrendre transform of minus the logarithm of Laplace transform” (also known as the Cramer transform) and that the logarithm of Laplace transform is connected to the cumulant-generating function.

β is a “geometric” (Planck) temperature, element of Lie algebra g of the group, and Q is a “geometric” heat, element of the dual space of the Lie algebra $g^{*}$ of the group in the Souriau Lie group thermodynamics model. The Riemannian metric proposed by Souriau has been identified by us as a generalization of the Fisher metric:

(9) $$I\left(\beta \right)=\left[g_{\beta }\right]\;\mathrm{with}\;g_{\beta }\left(\left[\beta ,Z_{1}\right],\left[\beta ,Z_{2}\right]\right)={\tilde{\Theta }}_{\beta }\left(Z_{1},\left[\beta ,Z_{2}\right]\right)$$

(10) $$\mathrm{with}\;{\tilde{\Theta }}_{\beta }\left(Z_{1},Z_{2}\right)=\tilde{\Theta }\left(Z_{1},Z_{2}\right)+\langle Q,ad_{Z_{1}}(Z_{2})\;\rangle \;\mathrm{where}\;ad_{Z_{1}}(Z_{2})=\left[Z_{1},Z_{2}\right]$$

Souriau proved that all coadjoint orbits of a Lie group given by $O_{F}=\left\{Ad_{g}^{*}F,g\in G\right\}\mathrm{subset}\;\mathrm{of}\;g^{*},F\in g^{*}$, carry, by a closed G-invariant 2-form, a natural homogeneous symplectic structure. If we define $K=Ad_{g}^{*}={\left(Ad_{g^{-1}}\right)}^{*}$ and $K_{*}(X)=-{\left(ad_{X}\right)}^{*}$ with:

(11) $$\langle Ad_{g}^{*}F,Y\rangle =\langle F,Ad_{g^{-1}}Y\rangle ,\forall g\in G,Y\in g,F\in g^{*}$$

where if X∈g,$Ad_{g}(X)=gXg^{-1}\in g$, the G-invariant 2-form is given by the following expression:

(12) $$\sigma_{\Omega }\left(ad_{X}F,ad_{Y}F\right)=B_{F}\left(X,Y\right)=\langle F,\left[X,Y\right]\rangle ,X,Y\in g$$

When a Lie group acts transitively by a Hamiltonian action on a symplectic manifold, the symplectic manifold is a covering space of a coadjoint orbit, according to Souriau’s fundamental theorem. We can see that the Fisher metric, in the non-equivariant situation, is an extension of this 2-form for the Souriau model:

(13) $$g_{\beta }\left(\left[\beta ,Z_{1}\right],\left[\beta ,Z_{2}\right]\right)=\tilde{\Theta }\left(Z_{1},\left[\beta ,Z_{2}\right]\right)+\langle Q,\left[Z_{1},\left[\beta ,Z_{2}\right]\right]\;\rangle$$

The non-equivariance induced an additional term by a symplectic cocycle, which corresponds to $\tilde{\Theta }\left(Z_{1},\left[\beta ,Z_{2}\right]\right)$. To define this extended Fisher metric, the tensor $\tilde{\Theta }$ used is defined by the moment map J(x), application from M (homogeneous symplectic manifold) to the dual space of the Lie algebra $g^{*}$, given by:

(14) $$\tilde{\Theta }(X,Y)=J_{\left[X,Y\right]}-\left\{J_{X},J_{Y}\right\}$$

$$\mathrm{with}\;J(x):M\to g^{*}\;\mathrm{such}\;\mathrm{that}\;J_{X}(x)=\langle J(x),X\rangle ,\;X\in g$$

As the tangent space of the cocycle $\theta \left(g\right)\in g^{*}$, $\tilde{\Theta }$ could be derived (the non-equivariance of the coadjoint operator $Ad_{g}^{*}$ generates this cocycle that modifies the action of the group on the dual space of the Lie algebra, so that the moment map could recover an equivariant relative to this new affine action):

(15) $$Q\left(Ad_{g}(\beta )\right)=Ad_{g}^{*}(Q)+\theta \left(g\right)$$

The cocycle $\theta \left(g\right)\in g^{*}$ is a measure characterizing the lack of equivariance of the moment map.

(16) $$\begin{array}{l} \tilde{\Theta }\left(X,Y\right):g\times g\to ℜ\;\;\;\mathrm{with}\;\Theta (X)=T_{e}\theta \left(X(e)\right)\\ \;\;\;\;\;\;X,Y\mapsto \langle \Theta (X),Y\rangle \end{array}$$

Souriau has then defined a Gibbs density that is covariant under the action of the group:

(17) $$p_{Gibbs}(\xi )=e^{\Phi (\beta )-\langle U(\xi ),\beta \rangle }=\frac{e^{-\langle U(\xi ),\beta \rangle }}{\int_{M}e^{-\langle U(\xi ),\beta \rangle }d\lambda_{\omega }}\;\mathrm{with}\;\Phi (\beta )=-\log\int_{M}e^{-\langle U(\xi ),\beta \rangle }d\lambda_{\omega }$$

(18) $$Q=\frac{\partial \Phi (\beta )}{\partial \beta }=\frac{\int_{M}U(\xi )e^{-\langle U(\xi ),\beta \rangle }d\lambda_{\omega }}{\int_{M}e^{-\langle U(\xi ),\beta \rangle }d\lambda_{\omega }}=\int_{M}U(\xi )p(\xi )d\lambda_{\omega }$$

We can express the Gibbs density with respect to Q by inverting the relation: $Q=\frac{\partial \Phi (\beta )}{\partial \beta }=\Theta \left(\beta \right)$. Then,

(19) $$p_{Gibbs,Q}(\xi )=e^{\Phi (\beta )-\langle U(\xi ),\Theta^{-1}\left(Q\right)\rangle }\mathrm{with}\;\beta =\Theta^{-1}\left(Q\right)$$

Souriau entropy $S\left(Q\right)$ is found to be constant on the affine coadjoint orbit of the group (where the “geometric heat” Q is an element of the dual space of the Lie algebra $g^{*}$ of the group) by observing that $S\left(Ad_{g}^{\#}(Q)\right)=S\left(Q\right)$ if we note the affine coadjoint operator $Ad_{g}^{\#}(Q)=Ad_{g}^{*}(Q)+\theta \left(g\right)$ where $\theta \left(g\right)$ is called the Souriau cocycle, and is associated with the default of equivariance of the moment map. We will next introduce this invariant Casimir function in coadjoint representation as the entropy within the context of Souriau Lie group thermodynamics. A function on M is a Casimir function when M is a Poisson manifold if and only if this function is constant on every symplectic leaf. Entropy is traditionally defined by Shannon with an axiomatic approach. Entropy will be defined in this essay as the Casimir equation’s solution for affine equivariance by:

(20) $${\left(ad_{\frac{\partial S}{\partial Q}}^{*}Q\right)}_{j}+\Theta {\left(\frac{\partial S}{\partial Q}\right)}_{j}=C_{ij}^{k}ad_{{\left(\frac{\partial S}{\partial Q}\right)}^{i}}^{*}Q_{k}+\Theta_{j}=0$$

where $\Theta (X)=T_{e}\theta \left(X(e)\right)\;\mathrm{with}\;\tilde{\Theta }\left(X,Y\right)=\langle \Theta (X),Y\rangle =J_{\left[X,Y\right]}-\left\{J_{X},J_{Y}\right\}$ in the non-null cohomology case (non-equivariance of coadjoint operator on the moment map), with $\theta \left(g\right)$ the Souriau symplectic cocycle. The Koszul–Fisher metric will be connected to the KKS 2-form, which links a homogeneous symplectic manifold structure to coadjoint orbits. In the context of thermodynamics, the fact that motion transverse to these surfaces is dissipative while motion remaining on them is non-dissipative could be used to explain how the information manifold foliates into level sets of entropy. 

$\frac{dQ}{dt}=ad_{\frac{\partial H}{\partial Q}}^{*}Q+\Theta \left(\frac{\partial H}{\partial Q}\right)$ with stable equilibrium given when $H=S\Rightarrow \frac{dQ}{dt}=ad_{\frac{\partial S}{\partial Q}}^{*}Q+\Theta \left(\frac{\partial S}{\partial Q}\right)=0$ (algorithm described preserves coadjoint orbits and Casimirs of the Lie–Poisson equation by construction). This equation could be written as the Euler–Poincaré equation [105].

We will also observe that $dS={\tilde{\Theta }}_{\beta }\left(\frac{\partial H}{\partial Q},\beta \right)dt$, where ${\tilde{\Theta }}_{\beta }\left(\frac{\partial H}{\partial Q},\beta \right)=\tilde{\Theta }\left(\frac{\partial H}{\partial Q},\beta \right)+\langle Q,\left[\frac{\partial H}{\partial Q},\beta \right]\;\rangle$, showing that the second law of thermodynamics could be deduced from the Souriau tensor positive definiteness related to Fisher–Koszul information metric. We can also extend the **affine Lie–Poisson equation** $\frac{dQ}{dt}=ad_{\frac{\partial H}{\partial Q}}^{*}Q+\Theta \left(\frac{\partial H}{\partial Q}\right)$**for stochastic dynamics**by a Stratonovich-kind differential equation given by:

(21) $$dQ+\left[ad_{\frac{\partial H}{\partial Q}}^{*}Q+\Theta \left(\frac{\partial H}{\partial Q}\right)\right]dt+\sum_{i=1}^{N}\left[ad_{\frac{\partial H_{i}}{\partial Q}}^{*}Q+\Theta \left(\frac{\partial H_{i}}{\partial Q}\right)\right]\circ dW_{i}\left(t\right)=0$$

## 4. Fisher Metric Symplectic Structure and Souriau–Casimir Entropy

We will give a definition of entropy purely geometrically based on this model as an extended invariant Casimir function defined on coadjoint orbits, where the cocycle characterizes the lack of equivariance for the moment mapping. The coadjoint orbits, which are also the entropy level sets, can be explained in terms of thermodynamics by the fact that dissipative phenomena are given by transverse dynamics to the symplectic leaves while non-dissipative ones are characterized by dynamics on the symplectic leaves. Additionally, by extending the Fisher–Koszul metric extension from information geometry by the Souriau tensor, the second thermodynamics law will be explained. We will also develop a new geometric heat equation using this Fisher–Koszul–Souriau tensor. 

### 4.1. Symplectic Structures of Fisher–Souriau Metric

Based on the seminal work of François Gallissot [106] developed by Souriau, in the “Lie group thermodynamics” model, β is a “geometric” temperature of Planck, an element of Lie algebra g of the group, and Q is a heat defined geometrically as an element of the dual space of the Lie algebra $g^{*}$ of the group. A Riemannian metric has been proposed by Souriau that we have identified as a generalization of the Fisher metric:

(22) $$I\left(\beta \right)=\left[g_{\beta }\right]\;\mathrm{with}\;g_{\beta }\left(\left[\beta ,Z_{1}\right],\left[\beta ,Z_{2}\right]\right)={\tilde{\Theta }}_{\beta }\left(Z_{1},\left[\beta ,Z_{2}\right]\right)$$

(23) $$\mathrm{with}\;{\tilde{\Theta }}_{\beta }\left(Z_{1},Z_{2}\right)=\tilde{\Theta }\left(Z_{1},Z_{2}\right)+\langle Q,ad_{Z_{1}}(Z_{2})\rangle \;\mathrm{where}\;ad_{Z_{1}}(Z_{2})=\left[Z_{1},Z_{2}\right]$$

where a Lie group acts transitively on a symplectic manifold by a Hamiltonian action, and the symplectic manifold is a covering space of a coadjoint orbit. We can see that in the non-equivariant case, the Fisher metric for the Souriau model is an extension of this 2-form $g_{\beta }\left(\left[\beta ,Z_{1}\right],\left[\beta ,Z_{2}\right]\right)=\tilde{\Theta }\left(Z_{1},\left[\beta ,Z_{2}\right]\right)+\langle Q,\left[Z_{1},\left[\beta ,Z_{2}\right]\right]\rangle$.

Due to the non-equivariance of the coadjoint operator induced by the symplectic cocycle, the term $\tilde{\Theta }\left(Z_{1},\left[\beta ,Z_{2}\right]\right)$ appears. To define the extended Fisher–Souriau metric, the tensor $\tilde{\Theta }$ could be deduced from the moment map J(x), application from the homogeneous symplectic manifold Mto the dual space of the Lie algebra $g^{*}$, given by:

(24) $$\tilde{\Theta }\left(X,Y\right)=J_{\left[X,Y\right]}-\left\{J_{X},J_{Y}\right\}$$

(25) $$\mathrm{with}\;J(x):M\to g^{*}\;\mathrm{such}\;\mathrm{that}\;J_{X}(x)=\langle J(x),X\rangle ,X\in g$$

**Figure 1:**
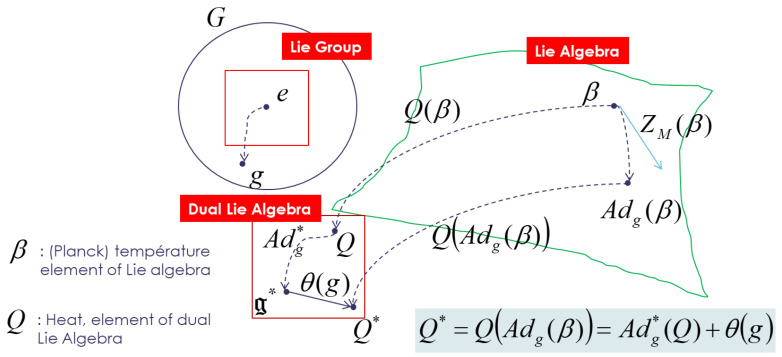
Lie group thermodynamics equations given by Souriau cocycle of affine equivariance in case of non-null cohomology.

As tangent space of the cocycle $\theta (g)\in g^{*}$, this tensor $\tilde{\Theta }$ could be introduced due to the non-equivariance of the coadjoint operator $Ad_{g}^{*}$ and the cocycle; we modify the action of the group on the dual space of the Lie algebra by adding a cocycle to introduce an “affine” equivariance of the momentum map as described in Figure 1:

(26) $$Q\left(Ad_{g}\left(\beta \right)\right)=Ad_{g}^{*}\left(Q\right)+\theta (g)$$

I use notation $Ad_{g}^{*}={\left(Ad_{g^{-1}}\right)}^{*}$ with $\langle Ad_{g}^{*}F,Y\rangle =\langle F,Ad_{g^{-1}}Y\rangle ,\forall g\in G,Y\in g,F\in g^{*}$ as used by Koszul and Souriau. $\theta (g)\in g^{*}$ is called non-equivariance 1-cocycle, and it is a measure of the lack of equivariance of the moment map.

(27) $$\begin{array}{ll} \tilde{\Theta }\left(X,Y\right): & g\times g\to ℜ\\ & X,Y\mapsto \tilde{\Theta }\left(X,Y\right)=\langle \Theta (X),Y\rangle \;\mathrm{with}\;\Theta (X)=T_{e}\theta \left(X(e)\right) \end{array}$$

It can be then deduced that the tensor could be also written by (with cocycle relation):

(28) $$\tilde{\Theta }\left(X,Y\right)=J_{\left[X,Y\right]}-\left\{J_{X},J_{Y}\right\}=-\langle d\theta (X),Y\rangle \;,\;X,Y\in g$$

(29) $$\tilde{\Theta }(\left[X,Y\right],Z)+\tilde{\Theta }(\left[Y,Z\right],X)+\tilde{\Theta }(\left[Z,X\right],Y)=0\;,\;X,Y,Z\in g$$

The bedrock of Souriau’s “Lie group thermodynamics” is given by the affine equivariance of the moment map J, characterized by an affine action of *G* on $g^{*}$, where we can recognize the classical coadjoint action as the linear part, for which the moment J is equivariant. When an element of the group g acts on the element β∈g of the Lie algebra, given by adjoint operator $Ad_{g}$ and with respect to the action of the group $Ad_{g}(\beta )$, the entropy $S\left(Q\right)$ and the Fisher metric $I\left(\beta \right)$ are invariant:

(30) $$\beta \in g\to Ad_{g}\left(\beta \right)\Rightarrow \left\{\begin{array}{l} S\left[Q\left(Ad_{g}\left(\beta \right)\right)\right]=S\left(Q\right)\\ I\left[Ad_{g}\left(\beta \right)\right]=I\left(\beta \right) \end{array}\right.$$

A 2-form in the Lie algebra has been introduced by Souriau, to deduce a Riemannian metric tensor in the values of the adjoint orbit of β, $\left[\beta ,Z\right]$ with Z an element of the Lie algebra. This metric is given for $\left(\beta ,Q\right)$:

(31) $$g_{\beta }\left(\left[\beta ,Z_{1}\right],\left[\beta ,Z_{2}\right]\right)=\langle \Theta \left(Z_{1}\right),\left[\beta ,Z_{2}\right]\rangle +\langle Q,\left[Z_{1},\left[\beta ,Z_{2}\right]\right]\rangle$$

where Θ is a cocycle of the Lie algebra, defined by $\Theta =T_{e}\theta$, with θ being a cocycle of the Lie group defined by $\theta \left(M\right)=Q\left(Ad_{M}\left(\beta \right)\right)-Ad_{M}^{*}Q$. The Riemannian metric given by Souriau based on his cocycle is a generalization of the Fisher–Koszul metric because we can define this metric as a Hessian of the partition function logarithm $g_{\beta }=-\frac{\partial^{2}\Phi }{\partial \beta^{2}}=\frac{\partial^{2}\log\psi_{\Omega }}{\partial \beta^{2}}$ as in classical information geometry. The equality of the two metrics could be proved by identifying the expression given by cocycle Θ and parameterized by *Q* (element of the dual space of the Lie algebra) and *β* (element of Lie algebra) with the Hessian of characteristic function $\Phi \left(\beta \right)=-\log\psi_{\Omega }\left(\beta \right)$ with respect to the variable *β*:

(32) $$g_{\beta }\left(\left[\beta ,Z_{1}\right],\left[\beta ,Z_{2}\right]\right)=\langle \Theta \left(Z_{1}\right),\left[\beta ,Z_{2}\right]\rangle +\langle Q,\left[Z_{1},\left[\beta ,Z_{2}\right]\right]\rangle =\frac{\partial^{2}\log\psi_{\Omega }}{\partial \beta^{2}}$$

If one assumes that $U\left(g\xi \right)=Ad_{g}^{*}U\left(\xi \right)+\theta_{\mu }\left(g\right),g\in G,\xi \in M$ which means that the energy $U:M\to g^{*}$ satisfies the same equivariance condition as the moment map $\mu :M\to g^{*}$, then one has for g∈G and β∈Ω

(33) $$\begin{array}{l} \Psi_{\Omega }\left(Ad_{g}\beta \right)=\int_{M}e^{-\langle U\left(\xi \right),Ad_{g}\beta \rangle }d\lambda (\xi )=\int_{M}e^{-\langle Ad_{g^{-1}}^{*}U\left(\xi \right),\beta \rangle }d\lambda (\xi )\\ =\int_{M}e^{-\langle U\left(g^{-1}\xi \right)-\theta_{\mu }(g^{-1}),\beta \rangle }d\lambda (\xi )=e^{\langle \theta_{\mu }(g^{-1}),\beta \rangle }\int_{M}e^{-\langle U\left(g^{-1}\xi \right),\beta \rangle }d\lambda (\xi )\\ =e^{\langle \theta_{\mu }(g^{-1}),\beta \rangle }\Psi_{\Omega }(\beta )\\ \Phi \left(Ad_{g}\beta \right)=-\log\Psi_{\Omega }\left(Ad_{g}\beta \right)=\Phi (\beta )-\langle \theta_{\mu }(g^{-1}),\beta \rangle \end{array}$$

To consider the invariance of entropy, we have to use the property that

(34) $$Q\left(Ad_{g}\beta \right)=Ad_{g}^{*}Q(\beta )+\theta_{\mu }(g)=g.Q(\beta )\;,\;\beta \in \Omega ,g\in G$$

For β∈Ω, let $g_{\beta }$ be the Hessian form on $T_{\beta }\Omega \equiv g$ with the potential $\Phi (\beta )=-\log\psi_{\Omega }(\beta )$. For X,Y∈g, we define:

(35) $$g_{\beta }\left(X,Y\right)=-\frac{\partial^{2}\Phi }{\partial \beta^{2}}\left(X,Y\right)={\left(\frac{\partial^{2}}{\partial s\partial t}\right)}_{s=t=0}\log\psi_{\Omega }\left(\beta +sX+tY\right)$$

The Cauchy–Schwarz inequality proves the positive definitiveness of this tensor.

We observe that $g_{\beta }\left(X,X\right)=0$ if and only if $\langle U\left(\xi \right),X\rangle$ is independent of ξ∈M, which means that the set $\left\{U\left(\xi \right);\xi \in M\right\}$ is contained in an affine hyperplane in $g^{*}$ perpendicular to the vector X∈g. We have seen that $g_{\beta }=-\frac{\partial^{2}\Phi }{\partial \beta^{2}}$, which is a **generalization of the classical Fisher metric from information geometry**, and will give the relation the Riemannian metric introduced by Souriau.

(36) $$g_{\beta }\left(X,Y\right)=\langle -\frac{\partial Q}{\partial \beta }(X),Y\rangle \;\mathrm{for}\;X,Y\in g$$

we have for any β∈Ω,g∈G and Y∈g:

(37) $$\langle Q\left(Ad_{g}\beta \right),Y\rangle =\langle Q(\beta ),Ad_{g^{-1}}Y\rangle +\langle \theta_{\mu }(g),Y\rangle$$

Let us differentiate the above expression with respect to g. Namely, we substitute $g=\exp\left(tZ_{1}\right),t\in R$ and differentiate at *t* = 0. Then the left-hand side of (22) becomes

(38) $${\left(\frac{d}{dt}\right)}_{t=0}\langle Q\left(\beta +t\left[Z_{1},\beta \right]+o\left(t^{2}\right)\right),Y\rangle =\langle \frac{\partial Q}{\partial \beta }\left(\left[Z_{1},\beta \right]\right),Y\rangle$$

and the right-hand side of (22) is calculated as:

(39) $$\begin{array}{l} {\left(\frac{d}{dt}\right)}_{t=0}\langle Q\left(\beta \right),Y-t\left[Z_{1},Y\right]+o\left(t^{2}\right)\rangle +\langle \theta_{\mu }\left(I+tZ_{1}+o\left(t^{2}\right)\right),Y\rangle \\ =-\langle Q(\beta ),\left[Z_{1},Y\right]\rangle +\langle d\theta_{\mu }\left(Z_{1}\right),Y\rangle \end{array}$$

Therefore,

(40) $$\langle \frac{\partial Q}{\partial \beta }\left(\left[Z_{1},\beta \right]\right),Y\rangle =\langle d\theta_{\mu }\left(Z_{1}\right),Y\rangle -\langle Q(\beta ),\left[Z_{1},Y\right]\rangle$$

Substituting $Y=-\left[\beta ,Z_{2}\right]$ to the above expression:

(41) $$\begin{array}{l} g_{\beta }\left(\left[\beta ,Z_{1}\right],\left[\beta ,Z_{2}\right]\right)=\langle -\frac{\partial Q}{\partial \beta }\left(\left[Z_{1},\beta \right]\right),\left[\beta ,Z_{2}\right]\rangle \\ g_{\beta }\left(\left[\beta ,Z_{1}\right],\left[\beta ,Z_{2}\right]\right)=-\langle d\theta_{\mu }(Z_{1}),\left[\beta ,Z_{2}\right]\rangle +\langle Q(\beta ),\left[Z_{1},\left[\beta ,Z_{2}\right]\right]\rangle \end{array}$$

We define then symplectic 2-cocycle and the tensor:

(42) $$\begin{array}{l} \Theta \left(Z_{1}\right)=-d\theta_{\mu }\left(Z_{1}\right)\\ \tilde{\Theta }\left(Z_{1},Z_{2}\right)=\langle \Theta \left(Z_{1}\right),Z_{2}\rangle =\left\{J_{Z_{1}},J_{Z_{2}}\right\}-J_{\left[Z_{1},Z_{2}\right]} \end{array}$$

Considering ${\tilde{\Theta }}_{\beta }\left(Z_{1},Z_{2}\right)=\langle Q\left(\beta \right),\left[Z_{1},Z_{2}\right]\rangle +\tilde{\Theta }\left(Z_{1},Z_{2}\right)$ as KKS 2-form when we have the property of non-null cohomology, we can then define the Fisher–Souriau metric:

(43) $$g_{\beta }\left(\left[\beta ,Z_{1}\right],\left[\beta ,Z_{2}\right]\right)={\tilde{\Theta }}_{\beta }\left(Z_{1},\left[\beta ,Z_{2}\right]\right)$$

A dual metric of the Fisher–Souriau metric is also given by entropy $S\left(Q\right)$ Hessian with respect to the dual variable given by *Q* due to the fact that the entropy is defined by the Legendre transform of the characteristic function, $\frac{\partial^{2}S\left(Q\right)}{\partial Q^{2}}$. 

It is an extension of the concept of “heat capacity.” Souriau called it a “geometric capacity”:

(44) $$I(\beta )=-\frac{\partial^{2}\Phi (\beta )}{\partial \beta^{2}}=-\frac{\partial Q}{\partial \beta }$$

**Figure 2:**
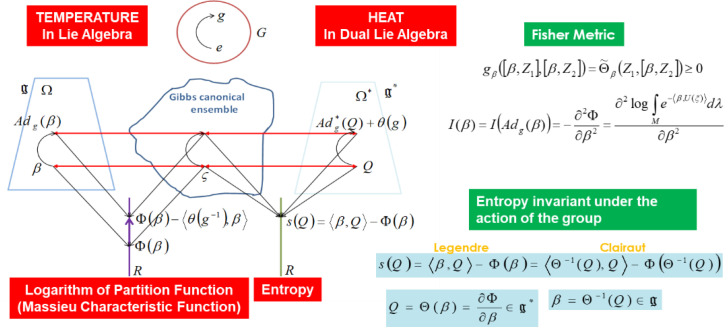
Souriau Lie group thermodynamics model and new geometric definition of the Fisher metric.

In Figure 2, we present Souriau’s illustration of his model. We can see that Pierre Duhem established the foundations of thermodynamics based on capacity and its expansion in a brand-new theory known as “energetique” in the previous century.

For the Galilean group in classical mechanics, for an isolated mechanical system, we cannot define any equilibrium Gibbs state, due to the non-existence of the open subset of the Lie algebra associated with this Gibbs state. We have then to consider the 1-parameter subgroup of the Galilean group produced by an element β of Lie algebra to be thought of as the following set of matrices:

$$\exp(\tau \beta )=\left(\begin{array}{ccc} A(\tau ) & \vec{b}(\tau ) & \vec{d}(\tau )\\ 0 & 1 & \tau \varepsilon \\ 0 & 0 & 1 \end{array}\right)\;\mathrm{with}\;\left\{\begin{array}{l} A(\tau )=\exp\left(\tau j(\vec{\omega })\right)\;\mathrm{and}\;\vec{b}(\tau )=\left(\sum_{i=1}^{\infty }\frac{\tau^{i}}{i!}{\left(j(\vec{\omega })\right)}^{i-1}\right)\vec{\alpha }\\ \vec{d}(\tau )=\left(\sum_{i=1}^{\infty }\frac{\tau^{i}}{i!}{\left(j(\vec{\omega })\right)}^{i-1}\right)\vec{\delta }+\varepsilon \left(\sum_{i=2}^{\infty }\frac{\tau^{i}}{i!}{\left(j(\vec{\omega })\right)}^{i-2}\right)\vec{\alpha } \end{array}\right.$$

where $\beta =\left(\begin{array}{ccc} j\left(\vec{\omega }\right) & \vec{\alpha } & \vec{\delta }\\ 0 & 1 & \varepsilon \\ 0 & 0 & 0 \end{array}\right)\in g$.

We can recover the classical thermodynamics by considering the reciprocal formula as follows:

$$Q=\frac{\partial \Phi }{\partial \beta },\;\beta =\frac{\partial S}{\partial Q},\;S(Q)=\langle \frac{\partial \Phi }{\partial \beta },\beta \rangle -\Phi \;\mathrm{and}\;\Phi (\beta )=\langle Q,\frac{\partial S}{\partial Q}\rangle -S$$

The classical Boltzmann–Clausius entropy for classical thermodynamics is recovered if the Lie group is restricted to only time translation:

$$\left\{\begin{array}{l} \beta =\frac{\partial S}{\partial Q}\\ \beta =\frac{1}{T} \end{array}\right.\Rightarrow dS=\frac{dQ}{T}$$

For instance, we may easily obtain the covariant Gibbs density for the centrifuge dynamical system using the Souriau model (equilibrium of angular moment map will be established by viscosity). According to Roger Balian, “Angular momentum is transferred to the gas when molecules hit with rotating wall, changing the Maxwell distribution at every location. The walls serve as a reservoir for angular momentum. Their motion is defined by an angular velocity, and at equilibrium, the angular velocities of the fluid and the walls are equalized, exactly as the temperature is equalized through energy exchanges.” According to Roger Balian, in order to characterize the equilibrium of a centrifuge, we need two hyperparameters of Lagrange, one for the classical thermodynamic equilibrium with Planck temperature and the other for the angular momentum equilibrium. Souriau made the observation that these two hyperparameters could coexist in the same tensor, which is a component of the Lie algebra of the Lie group acting on the system, and he defined what he termed a “geometric temperature” with a new ontology and a strictly geometric definition derived from symmetries.

**Figure 3:**
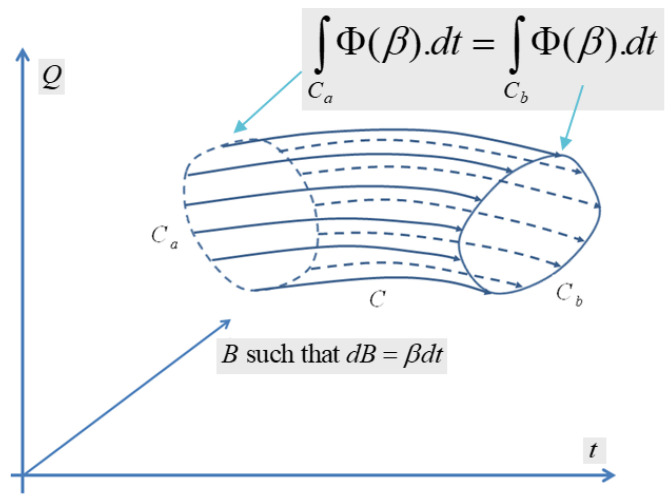
Souriau Lie group thermodynamics variational model and Souriau–Poincaré–Cartan integral invariant with respect to Massieu characteristic function.

We can see that by applying Poincaré–Cartan integral invariant [107] to the Massieu characteristic function, it is possible to derive generalized variational principles from the Souriau Lie group thermodynamics model, as illustrated in Figure 3. For the Souriau model, an extension of the Poincaré-Cartan integral invariant is provided by $\omega =\langle Q,\left(\beta .dt\right)\rangle -S.dt=\left(\langle Q,\beta \rangle -S\right).dt=\Phi (\beta ).dt$ with $g(t)\in G\;\mathrm{and}\;\beta (t)=g{\left(t\right)}^{-1}\dot{g}(t)\in g$. The variational model for an arbitrary path is $\delta \beta =\dot{\eta }+\left[\beta ,\eta \right]\;\mathrm{and}\;\delta \int_{t_{0}}^{t_{1}}\Phi \left(\beta (t)\right).dt=0$.

### 4.2. Souriau–Casimir Entropy and Lie Algebra Cohomology

Souriau’s equation $\langle Q,\left[\beta ,Z\right]\rangle +\tilde{\Theta }\left(\beta ,Z\right)=0$, introduced in 1974, can be proved by considering the curve $t\mapsto Ad_{\exp\left(tZ\right)}\beta \;\mathrm{with}\;Z\in g\;\mathrm{and}\;t\in R$. The curve $Ad_{\exp\left(tZ\right)}\beta$ passes, for t=0, through the point β, since $Ad_{\exp\left(0\right)}$ is the map for the identity of the Lie algebra g. This curve is in the adjoint orbit of β. So, by taking its derivative with respect to t, for t=0, a tangent vector in β is deduced at the adjoint orbit of this point. When Z takes arbitrary values in g, the vectors generate all the vector space tangent in β to the orbit of this point:

(45) $${\left.\frac{d\Phi \left(Ad_{\exp\left(tZ\right)}\beta \right)}{dt}\right|}_{t=0}=\langle \frac{d\Phi }{d\beta },\left({\left.\frac{d\left(Ad_{\exp\left(tZ\right)}\beta \right)}{dt}\right|}_{t=0}\right)\rangle =\langle Q,ad_{Z}\beta \rangle =\langle Q,\left[Z,\beta \right]\rangle$$

As we have seen before, $\Phi \left(Ad_{g}\beta \right)=\Phi \left(\beta \right)-\langle \theta \left(g^{-1}\right),\beta \rangle$. If we set $g=\exp\left(tZ\right)$, we obtain $\Phi \left(Ad_{\exp\left(tZ\right)}\beta \right)=\Phi \left(\beta \right)-\langle \theta \left(\exp\left(-tZ\right)\right),\beta \rangle$, and by derivation with respect to t at t=0, we finally recover the equation given by Souriau:

(46) $${\left.\frac{d\Phi \left(Ad_{\exp\left(tZ\right)}\beta \right)}{dt}\right|}_{t=0}=\langle Q,\left[Z,\beta \right]\rangle =-\langle d\theta (-Z),\beta \rangle \;\mathrm{with}\;\tilde{\Theta }\left(X,Y\right)=-\langle d\theta (X),Y\rangle$$

Souriau has developed this equation in greater depth. Souriau has just observed the identity $S\left[Q\left(Ad_{g}\left(\beta \right)\right)\right]=S\left[Ad_{g}^{*}\left(Q\right)+\theta (g)\right]=S\left(Q\right)$. We propose from this property to characterize this invariance more explicitly by **characterizing entropy as an invariant Casimir function in coadjoint representation**. From the last Souriau equation, if we use the identities $\beta =\frac{\partial S}{\partial Q}$, $ad_{\beta }Z=\left[\beta ,Z\right]$ and $\tilde{\Theta }\left(\beta ,Z\right)=\langle \Theta \left(\beta \right),Z\rangle$, then we can deduce that $\langle ad_{\frac{\partial S}{\partial Q}}^{*}Q+\Theta \left(\frac{\partial S}{\partial Q}\right),Z\rangle =0\;,\;\forall Z$. So, entropy $S\left(Q\right)$ should verify $ad_{\frac{\partial S}{\partial Q}}^{*}Q+\Theta \left(\frac{\partial S}{\partial Q}\right)=0$**,** which characterizes **an invariant Casimir function in the case of non-null cohomology** that we propose to write with Poisson brackets, where

(47) $${\left\{S,H\right\}}_{\tilde{\Theta }}\left(Q\right)=\langle Q,\left[\frac{\partial S}{\partial Q},\frac{\partial H}{\partial Q}\right]\rangle +\tilde{\Theta }\left(\frac{\partial S}{\partial Q},\frac{\partial H}{\partial Q}\right)=0\;,\;\forall H:g^{*}\to R\;,\;Q\in g^{*},\;$$

In fact, the following differentiation describes infinitesimal variation: $\frac{d}{dt}S{\left.\left(Q\left(Ad_{\exp\left(tx\right)}\beta \right)\right)\right|}_{t=0}=\frac{d}{dt}S{\left.\left(Ad_{\exp\left(tx\right)}^{*}Q+\theta \left(\exp\left(tx\right)\right)\right)\right|}_{t=0}=-\langle ad_{\frac{\partial S}{\partial Q}}^{*}Q+\Theta \left(\frac{\partial S}{\partial Q}\right),x\rangle$. When non-null cohomology occurs, we recover the equation $ad_{\frac{\partial S}{\partial Q}}^{*}Q+\Theta \left(\frac{\partial S}{\partial Q}\right)=0$ characterizing an invariant Casimir function, recovering the classical Casimir condition ${\left\{S,H\right\}}_{\tilde{\Theta }}\left(Q\right)=0$. The Lie–Poisson equations with cocycle solutions provide the Hamiltonian motion on these affine coadjoint orbits. 

Because entropy is traditionally introduced axiomatically, it appears as disruptive in information theory to identify entropy as a Casimir function constant on the coadjoint orbits. We are then able to build the entropy by an equation characterizing the Casimir function.

Because it adds a new differential equation structure in the situation of non-null cohomology, this new equation is significant. The variational principle modified for Lie–Poisson structure is equivalent to the prior Lie–Poisson equation:

(48) $$\delta \int_{0}^{\tau }\left(\langle Q(t),\frac{\partial H}{\partial Q}(t)\rangle -H\left(Q(t)\right)\right)dt=0\;\mathrm{where}\;\left\{\begin{array}{l} \frac{\partial H}{\partial Q}=g^{-1}\dot{g}\in g,g\in G\\ \frac{\partial^{2}H}{\partial Q^{2}}\delta Q=\delta \left(\frac{\partial H}{\partial Q}\right),\eta =g^{-1}\delta g\\ \langle Q,\delta \left(\frac{\partial H}{\partial Q}\right)\rangle =\langle Q,\dot{\eta }+\left[\frac{\partial H}{\partial Q},\eta \right]\rangle +\tilde{\Theta }\left(\frac{\partial H}{\partial Q},\eta \right) \end{array}\right.$$

**Geometric Definition of Heat Equation**

From this Lie–Poisson equation, we can introduce a ***geometric heat Fourier equation***:

(49) $$\frac{\partial Q}{\partial t}=ad_{\frac{\partial H}{\partial Q}}^{*}Q+\Theta \left(\frac{\partial H}{\partial Q}\right)\;\mathrm{and}\;\frac{\partial F\left(Q\right)}{\partial t}={\left\{F\left(Q\right),H\right\}}_{\tilde{\Theta }}$$

that we can rewrite:

(50) $$\frac{\partial Q}{\partial t}=\frac{\partial Q}{\partial \beta }\frac{\partial \beta }{\partial t}=ad_{\frac{\partial H}{\partial Q}}^{*}Q+\Theta \left(\frac{\partial H}{\partial Q}\right)$$

where $\frac{\partial Q}{\partial \beta }$ geometric heat capacity is given by $g_{\beta }\left(X,Y\right)=\langle -\frac{\partial Q}{\partial \beta }(X),Y\rangle \;\mathrm{for}\;X,Y\in g\;\mathrm{with}\;g_{\beta }\left(X,Y\right)={\tilde{\Theta }}_{\beta }\left(X,Y\right)=\langle Q\left(\beta \right),\left[X,Y\right]\rangle +\tilde{\Theta }\left(X,Y\right)$ related to Souriau-Fisher tensor.

The PDE for (calorific) energy density known as the heat equation uses the geometric heat capacity to describe the characteristics of a material. The method of numerically integrating Lie–Poisson systems while maintaining coadjoint orbits has been considered.

In the homogeneous Euclidean situation, we have the following classical equation:

(51) $$\frac{\partial \rho_{E}}{\partial t}=div\left(\frac{\lambda }{C}\nabla \rho_{E}\right)\mathrm{with}\;\frac{\partial \rho_{E}}{\partial t}=C\frac{\partial T}{\partial t}$$

The relationship with the **second law of thermodynamics** will be inferred from the Souriau–Fisher tensor positivity:

(52) $$\begin{array}{l} S\left(Q\right)=\langle Q,\beta \rangle -\Phi \left(\beta \right)\;\mathrm{with}\;\frac{dQ}{dt}=ad_{\frac{\partial H}{\partial Q}}^{*}Q+\Theta \left(\frac{\partial H}{\partial Q}\right)\\ \Rightarrow \frac{dS}{dt}={\tilde{\Theta }}_{\beta }\left(\frac{\partial H}{\partial Q},\beta \right)\geq 0,\forall H\;(\mathrm{link}\;\mathrm{to}\;\mathrm{positivity}\;\mathrm{of}\;\mathrm{Fisher}\;\mathrm{metric})\\ \mathrm{if}\;H=S\underset{\frac{\partial S}{\partial Q}=\beta }{\Rightarrow }\frac{dS}{dt}={\tilde{\Theta }}_{\beta }\left(\beta ,\beta \right)=0\;\mathrm{because}\;\beta \in Ker{\tilde{\Theta }}_{\beta } \end{array}$$

Entropy production is then linked with Souriau–Fisher structure, $dS={\tilde{\Theta }}_{\beta }\left(\frac{\partial H}{\partial Q},\beta \right)dt$ with ${\tilde{\Theta }}_{\beta }\left(\frac{\partial H}{\partial Q},\beta \right)=\tilde{\Theta }\left(\frac{\partial H}{\partial Q},\beta \right)+\langle Q,\left[\frac{\partial H}{\partial Q},\beta \right]\rangle$ Souriau–Fisher tensor.

The two equations characterizing entropy as an invariant Casimir function are related by:

(53) $$\forall H,{\left\{S,H\right\}}_{\tilde{\Theta }}\left(Q\right)=\langle ad_{\frac{\partial S}{\partial Q}}^{*}Q+\Theta \left(\frac{\partial S}{\partial Q}\right),\frac{\partial H}{\partial Q}\rangle =0\Rightarrow ad_{\frac{\partial S}{\partial Q}}^{*}Q+\Theta \left(\frac{\partial S}{\partial Q}\right)=0$$

This equation appears in the Souriau paper published in 1974, observing that geometric temperature β is a kernel of ${\tilde{\Theta }}_{\beta }$, which is written as follows:

(54) $$\beta \in Ker{\tilde{\Theta }}_{\beta }\Rightarrow \langle Q,\left[\beta ,Z\right]\rangle +\tilde{\Theta }\left(\beta ,Z\right)=0$$

and can be developed to recover the Casimir equation:

(55) $$\begin{array}{l} \Rightarrow \langle Q,ad_{\beta }Z\rangle +\tilde{\Theta }\left(\beta ,Z\right)=0\Rightarrow \langle ad_{\beta }^{*}Q,Z\rangle +\tilde{\Theta }\left(\beta ,Z\right)=0\\ \beta =\frac{\partial S}{\partial Q}\Rightarrow \langle ad_{\frac{\partial S}{\partial Q}}^{*}Q,Z\rangle +\tilde{\Theta }\left(\frac{\partial S}{\partial Q},Z\right)=\langle ad_{\frac{\partial S}{\partial Q}}^{*}Q+\Theta \left(\frac{\partial S}{\partial Q}\right),Z\rangle =0,\forall Z\\ \Rightarrow ad_{\frac{\partial S}{\partial Q}}^{*}Q+\Theta \left(\frac{\partial S}{\partial Q}\right)=0 \end{array}$$

### 4.3. Link between Souriau–Casimir Entropy and Koszul–Poisson Cohomology 

J.L. Koszul and A. Lichnerowicz and [108,109,110] introduced Poisson cohomology. Koszul cited a seminal paper by E. Cartan that stated, “Elie Cartan does not explicitly mention Λ(g’) [the complex of alternate forms on a Lie algebra], because he treats groups as symmetrical spaces and is interested in differential forms that are invariant to both the translations to the left and the translations to the right, which corresponds to the elements of Λ(g’) invariant by the prolongation of the coadjoint representation. Nevertheless, it can be claimed that a crucial aspect of the cohomological theory of Lie algebras was in place by 1929.” We can also make reference to F. Berezin [111]. 

When defining the Poisson structure, A. Lichnerowics pointed out that it can be written using the Schouten–Nijenhuis bracket [112,113,114,115,116] and that it is an extension of symplectic structures that use contravariant tensor fields rather than differential forms. Let us consider $ad_{P}Q=\left[P,Q\right]$, where $ad_{P}$ is a graded linear endomorphism of degree *p-1* of *A(M)*. From the graded Jacobi identity, we can write:

(56) $$\begin{array}{l} ad_{P}\left[Q,R\right]=\left[ad_{P}Q,R\right]+(-1){(}^{p}{-}^{1}{)}^{\;}{(}^{q}{-}^{1}{)}^{\;}\left[Q,ad_{p}R\right]\\ ad_{\left[P,Q\right]}=ad_{P}\circ ad_{Q}-(-1){(}^{p}{-}^{1}{)}^{\;}{(}^{q}{-}^{1}{)}^{\;}ad_{Q}\circ ad_{P} \end{array}$$

The first equation means that the graded endomorphisms $ad_{P}Q=\left[P,Q\right]$, of degree *p-1*, are a derivation of the graded Lie algebra *A(M)* with the **Schouten–Nijenhuis bracket** as composition law. The second equation of means that the endomorphism $ad_{\left[P,Q\right]}$ is the graded commutator of endomorphisms $ad_{P}$ and $ad_{Q}$. Y. Vorob’ev and M.V. Karasev have suggested cohomology classification in terms of closed forms and de Rham cohomology of coadjoint orbits Ω (called Euler orbits by authors), symplectic leaves of a Poisson manifold Ν. Let $Z^{k}\left(\Omega \right)$ and $H^{k}\left(\Omega \right)$ be the space of closed k-forms on Ω and their de Rham cohomology classes. Considering the base of the fibration of Ν by these orbits as Ν/Ω, they have introduced the map $Z^{k}\left[\Omega \right]=C^{\infty }\left(N/\Omega \to Z^{k}\left(\Omega \right)\right)$ and $H^{k}\left[\Omega \right]=C^{\infty }\left(N/\Omega \to H^{k}\left(\Omega \right)\right)$. Depending on coordinates on Ν/Ω, the elements of $Z^{k}\left[\Omega \right]$ are closed forms on Ω. **Then** $H^{0}\left[\Omega \right]=Casim\left(N\right)$ **is identified with the Casimir functions set on** Ν **of constant functions on all coadjoint orbits. Entropy is then given by zero-dimensional de Rham cohomology.** For arbitrary $v\in V\left(N\right)$, with the set $V\left(N\right)$ of all vector fields on N, the tensor Dv defines a closed 2-form $\alpha \left(Dv\right)=Z^{2}\left[\Omega \right]$, and if $v\in V\left(N\right)$ annihilates $H^{0}\left[\Omega \right]=Casim\left(N\right)$**,** then this form is exact. The center of Poisson algebra induced from the symplectic structure is the zero-dimensional de Rham cohomology group, the Casimir functions.

## 5. Metriplectic Model for Dissipative Heat Equation

A Hamiltonian model of dynamics is used to identify systems that maintain energy throughout the phase. Dissipative effects, which are irreversible changes from a thermodynamic perspective, cannot be accounted for in classical Hamiltonian systems. The metriplectic bracket was first introduced in 1983 by A. N. Kaufman and P. J. Morrison. This bracket formalism provides both energy conservation and a non-decrease in entropy, and it reduces to the traditional Poisson bracket formalism in the limit of no dissipation. Parallel axiomatization of this model was performed by Grmela and Öttinger (called GENERIC method: General Equation for Non-Equilibrium Reversible and Irreversible Coupling). 

There are three different types of dissipation: thermal diffusion with energy conservation and entropy production through heat transfer; viscosity, which takes energy from the system (e.g., Navier–Stokes equation); and transport equations with collision operators. These types of systems that adhere to both the first and second laws of thermodynamics are included in metriplectic dynamics. A new bracket in the metriplectic formalism provides the evolution equation $\left\{\left\{.,.\right\}\right\}$:

(57) $$\frac{df}{dt}=\left\{\left\{f,F\right\}\right\}=\left\{f,F\right\}+\left(f,F\right)$$

Hamiltonian components are introduced by requiring:

(58) F=H+S

The second bracket has two constraints:

(59) $$\left(f,F\right)=\left(F,f\right)\;\mathrm{and}\;\left(f,f\right)\geq 0$$

with the entropy S selected from the set of Casimir invariants of the noncanonical Poisson bracket, playing the role of a Lyapunov functional. A metriplectic vector field induced by F is given by the dynamics:

(60) $$\frac{dz_{i}}{dt}=J_{ij}\frac{\partial F}{\partial z_{j}}+M_{ij}\frac{\partial F}{\partial z_{j}}$$

compliant with two first principles of thermodynamics:

- First principle: energy conservation:

(61) $$\begin{array}{l} \frac{dH}{dt}=\left\{H,F\right\}+\left(H,F\right)=\left\{H,H\right\}+\left\{H,S\right\}+\left(H,H\right)+\left(H,S\right)=0\\ \mathrm{because}\;\left\{\begin{array}{l} \left\{H,H\right\}=0\;\mathrm{by}\;\mathrm{symmetry}\\ \left\{f,S\right\}=0,\forall f\\ \left(H,f\right)=0,\forall f \end{array}\right. \end{array}$$

- Second principle: entropy production:

(62) $$\begin{array}{l} \frac{dS}{dt}=\left\{S,F\right\}+\left(S,F\right)=0+\left(S,H\right)+\left(S,S\right)=\left(S,S\right)\geq 0\\ \mathrm{because}\;\left\{\begin{array}{l} \left\{S,f\right\}=0,\forall f\\ \left(f,H\right)=0,\forall f\\ M\;\mathrm{positive}\;\mathrm{semi}-\mathrm{definite}\;\Rightarrow \;\left(S,S\right)\geq 0 \end{array}\right. \end{array}$$

By using entropy as the Casimir invariant function, thermal equilibrium is recovered.

Finally, two compatible brackets—a Poisson bracket and a symmetric bracket—determine the geometry in metriplectic systems:

(63) $$\frac{df}{dt}=\left\{\left\{f,F\right\}\right\}=\left\{f,H\right\}+\left(f,S\right)$$

The energy *H* is a Casimir invariant of the dissipative bracket, and the entropy *S* is a Casimir invariant of the Poisson bracket:

(64) $$\begin{array}{l} \left\{S,H\right\}=0\forall H\\ \left(H,S\right)=0\forall S \end{array}$$

### 5.1. Canonical/Noncanonical Hamiltonian Structures and Poisson Bracket $\left\{.,.\right\}$

The first part of a metriplectic vector field relative to the non-dissipative part is given by the Poisson bracket. Considering the Hamiltonian function $H\left(q,p\right)$ depending on the canonical coordinates q and momenta p, with $z=\left(q,p\right)$, Hamiltonian equations:

(65) $$\frac{dq_{i}}{dt}=\frac{\partial H}{\partial p_{i}}\;\mathrm{and}\;\frac{dq_{i}}{dt}=-\frac{\partial H}{\partial q_{i}},i=1,\ldots ,N$$

can be rewritten in tensorial form with canonical Poisson matrix and bracket:

(66) $$\begin{array}{l} \frac{dz_{i}}{dt}=J_{ij}\frac{\partial H}{\partial z_{j}}=\left\{z_{i},H\right\},i,j=1,\ldots ,2N\\ J=\left(\begin{array}{cc} 0_{N} & I_{N}\\ -I_{N} & 0_{N} \end{array}\right)\;\mathrm{and}\;\left\{f,g\right\}=\frac{\partial f}{\partial z_{j}}J_{ij}\frac{\partial g}{\partial z_{j}} \end{array}$$

The Poisson structure (tensor) needs not be nondegenerate. The only case where it is nondegenerate is the symplectic case, which we are considering here.

We note the symplectic 2-form $\omega =\sum_{}dp^{i}\wedge dq_{i}$ such that $\omega^{ik}J_{kj}=\delta_{ij}$.

By change of coordinates $\hat{z}=\hat{z}(z)$, the Poisson matrix J transforms as a contravariant 2-tensor:

(67) $${\hat{J}}_{ij}(\hat{z})=J_{uv}\frac{\partial {\hat{z}}_{i}}{\partial z_{u}}\frac{\partial {\hat{z}}_{j}}{\partial z_{v}}\;\mathrm{and}\;\frac{d{\hat{z}}_{i}}{dt}={\hat{J}}_{ij}\frac{\partial \hat{H}}{\partial {\hat{z}}_{j}}\;\mathrm{with}\;\hat{H}(\hat{z})=H(z)$$

If ${\hat{J}}_{ij}=J_{ij}$, the transformation is called a canonical transformation or symplectomorphism, and Hamilton’s equations are preserved:

(68) $$\frac{d{\hat{q}}_{i}}{dt}=\frac{\partial \hat{H}}{\partial {\hat{p}}_{i}}\;\mathrm{and}\;\frac{d{\hat{p}}_{i}}{dt}=-\frac{\partial \hat{H}}{\partial {\hat{q}}_{i}},i=1,\ldots ,N\;\mathrm{with}\;\hat{z}=\left(\hat{q},\hat{p}\right)$$

The noncanonical generalization of the Hamiltonian form is given by:

(69) $$\begin{array}{l} \frac{dz_{i}}{dt}=J_{ij}^{NC}\frac{\partial H}{\partial z_{j}}=\left\{z_{i},H\right\},i=1,\ldots ,M\\ \left\{f,g\right\}=\frac{\partial f}{\partial z_{i}}J_{ij}^{NC}\frac{\partial g}{\partial z_{j}},i,j=1,\ldots ,M \end{array}$$

For a Poisson bracket, we should have the following properties:

(70) $$\begin{array}{l} \mathrm{Antisymmetry}:\;\left\{f,g\right\}=-\left\{g,f\right\}\Leftrightarrow J^{ij}=-J^{ji}\\ \mathrm{Jacobi}\;\mathrm{Identity}:\;\left\{f,\left\{g,h\right\}\right\}+\left\{h,\left\{f,g\right\}\right\}+\left\{g,\left\{h,f\right\}\right\}=0 \end{array}$$

If $\det\left(J^{NC}\right)\neq 0$, then from the Darboux theorem there exists a coordinate change that at least locally brings $J^{NC}$ into the canonical form J:

(71) $$J_{uv}^{NC}\frac{\partial {\hat{z}}_{i}}{\partial z_{u}}\frac{\partial {\hat{z}}_{j}}{\partial z_{v}}=J_{ij}$$

If $\det\left(J^{NC}\right)=0$, there exist Casimir invariants C which are constants of motion for any Hamiltonian:

(72) $$\left\{f,C\right\}=0,\forall f\Leftrightarrow J_{ij}\frac{\partial C}{\partial z_{j}}=0,\forall i$$

The level sets of the Casimir invariants are the foliation of the phase space. An orbit initiated on the level sets of the initial Casimir invariants remains on these symplectic leaves.

If $\det\left(J^{NC}\right)=0$, from the Lie–Darboux theorem, there is no coordinate transformation to canonical form; however, there is a transformation to the following degenerate canonical form:

(73) $$J_{\mathrm{degenerate}}=\left(\begin{array}{ccc} 0_{N} & I_{N} & 0\\ -I_{N} & 0_{N} & 0\\ 0 & 0 & 0_{M-2N} \end{array}\right)$$

For finite-dimensional Lie–Poisson Hamiltonian systems, the Poisson matrix J is linear in the dynamical variable and has the form $J_{ij}=c_{ij}^{k}.z_{k}$, where the numbers $c_{ij}^{k}$ are the structure constants of some Lie algebra.

### 5.2. Metric Flow Structures and Symmetric Bracket $\left(.,.\right)$

A flow in metric space makes up the second component of a metriplectic vector field in relation to the dissipative component. The coordinate form of the metric flow on a finite-dimensional phase space manifold is as follows:

(74) $$\begin{array}{l} \frac{dz_{i}}{dt}=M_{ij}\frac{\partial S}{\partial z_{j}}=\left(z_{i},S\right),i=1,\ldots ,M\\ \left(f,g\right)=\frac{\partial f}{\partial z_{j}}M_{ij}(z)\frac{\partial g}{\partial z_{j}},i,j=1,\ldots ,M\;\mathrm{with}\;\left(f,g\right)=\left(g,f\right) \end{array}$$

where S is an entropy function. We should have the following properties for the metric:

(75) $$\begin{array}{l} M\;\mathrm{positive}\;\mathrm{semi}-\mathrm{definite}\;\Rightarrow \;\frac{dS}{dt}=\left(S,S\right)\geq 0\\ \mathrm{Energy}\;\mathrm{Conservation}\;H\;(\mathrm{Hamiltonian})\Rightarrow \;\left(H,f\right)=0,\forall f \end{array}$$

The symmetry requirement generalizes the Onsager symmetry of linear irreversible thermodynamics to nonlinear issues; however, in the traditional metriplectic model, the possibility of Casimir symmetry is not taken into account.

The double bracket, Cartan–Killing bracket and Casimir dissipation bracket are three dissipation brackets $\left(.,.\right)$ that have been presented in the literature for the metriplectic system in the Lie–Poisson framework. The Lie–Poisson bracket for two functions f and h in Lie–Poisson systems is provided by:

(76) $$\left\{f,h\right\}(z)=\langle z,\left[\frac{\partial f}{\partial z},\frac{\partial h}{\partial z}\right]\rangle =\langle z,-ad_{\frac{\partial H}{\partial z}}\frac{\partial f}{\partial z}\rangle =\langle ad_{\frac{\partial h}{\partial z}}^{*}z,\frac{\partial f}{\partial z}\rangle$$

Hamiltonian dynamics is given by:

(77) $$\frac{df}{dt}=\left\{f,h\right\}(z)=\langle ad_{\frac{\partial h}{\partial z}}^{*}z,\frac{\partial f}{\partial z}\rangle \Rightarrow \frac{dz}{dt}=ad_{\frac{\partial h}{\partial z}}^{*}z$$

In coordinate realization, with a coordinate chart $\left(z_{i}\right)$, the Poisson bivector is represented by a set of coefficient functions determining the Poisson bracket:

(78) $$\left\{f,h\right\}=J_{ij}\frac{\partial f}{\partial z_{i}}\frac{\partial h}{\partial z_{j}}\Rightarrow \frac{dz_{i}}{dt}=J_{ij}\frac{\partial h}{\partial z_{j}}$$

For Lie–Poisson structure defined on the dual of a finite-dimensional Lie algebra, we can introduce structure constants with an N-dimensional Lie algebra admitting a basis $\left\{e_{1},\ldots ,e_{N}\right\}$: $\left[e_{i},e_{j}\right]=c_{ij}^{k}e_{k}$ (with summation convention over the repeated indices). The Lie–Poisson dynamics is given by:

(79) $$J_{ij}=c_{ij}^{k}z_{k}\Rightarrow \frac{dz_{j}}{dt}=c_{ij}^{k}z_{k}\frac{\partial h}{\partial z_{i}}$$

#### 5.2.1. Dissipation Bracket as Double Bracket

The double bracket is given by:

(80) $$\left(f,h\right)=\sum_{j}J_{ij}J_{lj}\frac{\partial f}{\partial z_{i}}\frac{\partial h}{\partial z_{l}}=\sum_{j}c_{ij}^{k}c_{lj}^{r}z_{k}z_{r}\frac{\partial f}{\partial z_{i}}\frac{\partial f}{\partial z_{l}}$$

with the metriplectic dynamics:

(81) $$\frac{dz_{j}}{dt}=c_{ij}^{k}z_{k}\frac{\partial h}{\partial z_{i}}+\sum_{i}c_{ji}^{k}c_{li}^{r}z_{r}z_{n}\frac{\partial h}{\partial z_{l}}$$

#### 5.2.2. Dissipation bracket as Cartan–Killing Bracket

Given a basis $\left\{e_{1},\ldots ,e_{N}\right\}$ in an N-dimensional Lie algebra g, for any element X of g, we can decompose it by the structure constants of g, antisymmetric in their lower indices $c_{ij}^{k}=-c_{ji}^{k}$:

(82) $$X=\sum_{i=1}^{N}x_{i}e_{i}\;\mathrm{with}\;\left[e_{i},e_{j}\right]=c_{ij}^{k}e_{k}$$

and the linear operator:

(83) $$ad_{X}Z=\left[X,Z\right]=\sum x_{i}z_{j}c_{ij}^{k}e_{k}\;\mathrm{and}\;ad_{X}ad_{Y}Z=\left[X,\left[Y,Z\right]\right]=c_{il}^{k}c_{jm}^{l}x_{i}y_{j}z_{m}e_{k}$$

Taking the trace of the linear operator defines the Killing form, a symmetric bilinear form on vectors of the Lie algebra:

(84) $$\begin{array}{l} K\left(X,Y\right)=tr\left(ad_{X}ad_{Y}\right)=\sum_{i,j}c_{kj}^{i}c_{li}^{j}x_{k}y_{l}=g_{kl}x_{k}y_{l}\;\mathrm{with}\;g_{ij}=\sum_{k,l}c_{il}^{k}c_{jk}^{l}=tr\left(ad_{e_{i}}ad_{e_{j}}\right)\\ K\left(ad_{Z}X,Y\right)+K\left(X,ad_{Z}Y\right)=0 \end{array}$$

and the Jacobi identity is deduced from the following relation on the structure constants:

(85) $$\sum_{k}c_{ik}^{r}c_{jl}^{k}+c_{jk}^{r}c_{li}^{k}+c_{lk}^{r}c_{ij}^{k}=0\;\mathrm{also}\;\mathrm{deduced}\;\mathrm{from}\;\left[ad_{X},ad_{Y}\right]Z=ad_{\left[X,Y\right]}Z$$

The tensor $g_{ij}$ can be used to lower the third label of $c_{ij}^{k}$ by defining:

(86) $$\begin{array}{l} c_{ijk}=c_{ij}^{l}g_{kl}=c_{ij}^{l}c_{kr}^{m}c_{lm}^{r}\;\mathrm{is}\;\mathrm{completely}\;\mathrm{antisymmetric}\;\mathrm{due}\;\mathrm{to}\\ K\left(X,ad_{Y}Z\right)=K(Y,ad_{Z}X)=K\left(Z,ad_{X}Y\right)=c_{ijk}x_{i}y_{j}z_{k}=c_{jki}y_{j}z_{k}x_{i}=c_{kij}z_{k}x_{i}y_{j} \end{array}$$

Given a semisimple Lie algebra g, with an invertible Killing form, we define:

(87) $$C_{q}=\sum_{i,j}g^{ij}e_{i}e_{j}\;\mathrm{with}\;g_{ij}g^{jk}=\delta_{ij}$$

$C_{q}$, the quadratic Casimir operator, has a vanishing bracket with any $e_{i}$ and hence with any element of g:

(88) $$\begin{array}{l} \left[C_{q},e_{k}\right]=\sum_{i,j}g^{ij}\left[e_{i}e_{j},e_{k}\right]=\sum_{i,j}g^{ij}\left(e_{i}\left[e_{j},e_{k}\right]+\left[e_{i},e_{k}\right]e_{j}\right)\\ \left[C_{q},e_{k}\right]=\sum_{i,j,k}g^{ij}c_{jl}^{k}\left(e_{i}e_{k}+e_{k}e_{i}\right)=\sum_{i,j,k,r}g^{ij}g^{kr}c_{jlr}\left(e_{i}e_{k}+e_{k}e_{i}\right)=0 \end{array}$$

The term $\sum_{j,r}g^{ij}g^{kr}c_{jlr}$ is antisymmetric in *i* and *k*, while the term in parentheses is symmetric, and then the sum vanishes. For a simple Lie algebra, in its universal enveloping algebra, a quadratic expression in e that commutes with all the e’s is proportional to the Casimir operator. The quadratic Casimir operator is then unique up to a factor.

If we come back to the Cartan–Killing metric $g_{ij}=\sum_{k,l}c_{il}^{k}c_{jk}^{l}=tr\left(ad_{e_{i}}ad_{e_{j}}\right)$ that defines a symmetric and bilinear covariant tensor, a symmetric bracket for functions in terms of the metric is then given by:

(89) $$\left(f,h\right)=c_{il}^{k}c_{jk}^{l}\frac{\partial f}{\partial z_{i}}\frac{\partial f}{\partial z_{l}}$$

with the metriplectic dynamics:

(90) $$\frac{dz_{j}}{dt}=c_{ij}^{k}z_{k}\frac{\partial h}{\partial z_{i}}+c_{jr}^{i}c_{li}^{r}\frac{\partial h}{\partial z_{l}}$$

#### 5.2.3. Dissipation bracket as Casimir Dissipation Bracket

Given a Casimir function S(z), for $z\in g^{*}$, a positive symmetric bilinear form $\gamma_{z}$ and a real number θ>0, the Lie–Poisson dynamical equation is modified to produce the Casimir dissipative Lie–Poisson equation:

(91) $$\frac{df(z)}{dt}=\left\{f,h\right\}-\theta \gamma_{z}\left(\left[\frac{\partial f}{\partial z},\frac{\partial h}{\partial z}\right],\left[\frac{\partial S}{\partial z},\frac{\partial h}{\partial z}\right]\right)$$

inducing the following dynamical equation:

(92) $$\begin{array}{l} \frac{dz}{dt}=ad_{\frac{\partial h}{\partial z}}^{*}z+\theta ad_{\frac{\partial h}{\partial z}}^{*}{\left[\frac{\partial S}{\partial z},\frac{\partial h}{\partial z}\right]}^{b}=ad_{\frac{\partial h}{\partial z}}^{*}\left(z+\theta {\left[\frac{\partial S}{\partial z},\frac{\partial h}{\partial z}\right]}^{b}\right)\\ \mathrm{with}\;b:g\to g^{*}\;\mathrm{such}\;\mathrm{that}\;\xi \in g,\xi^{b}\in g^{*}\;\mathrm{then}\;\langle \xi^{b},\eta \rangle =\gamma_{z}\left(\xi ,\eta \right)\forall \eta \in g \end{array}$$

We can identify this model in the framework of metriplectic one by the following identification:

(93) $$-\theta \gamma_{z}\left(\left[\frac{\partial f}{\partial z},\frac{\partial h}{\partial z}\right],\left[\frac{\partial g}{\partial z},\frac{\partial h}{\partial z}\right]\right)=\theta \langle \frac{\partial f}{\partial z},ad_{\frac{\partial h}{\partial z}}^{*}\left({\left[\frac{\partial g}{\partial z},\frac{\partial h}{\partial z}\right]}^{b_{z}}\right)\rangle =\langle \frac{\partial f}{\partial z},\kappa_{z}\left(\frac{\partial g}{\partial z}\right)\rangle =\left(f,g\right)\forall g\in C^{\infty }\left(g^{*}\right)$$

#### 5.2.4. Hamilton Dissipation Bracket

Assuming a symmetric semi-positive definite bilinear operator ψ defined on a Lie algebra g, we fix a Casimir function S(z) of the Lie–Poisson bracket and introduce the following symmetric bracket on the dual space $g^{*}$, for two functionals f and h, given by:

(94) $$\left(f,h\right)=-\psi \left(\left[\frac{\partial f}{\partial z},\frac{\partial S}{\partial z}\right],\left[\frac{\partial h}{\partial z},\frac{\partial S}{\partial z}\right]\right)$$

The associated equation of motion is as follows:

(95) $$\begin{array}{l} \frac{dz}{dt}=ad_{\frac{\partial h}{\partial z}}^{*}z-ad_{\frac{\partial S}{\partial z}}^{*}{\left[\frac{\partial S}{\partial z},\frac{\partial h}{\partial z}\right]}^{b}\\ \mathrm{with}\;b:g\to g^{*}\;\mathrm{such}\;\mathrm{that}\;\xi \in g,\xi^{b}\in g^{*}\;\mathrm{then}\;\langle \xi^{b},\eta \rangle =\psi \left(\xi ,\eta \right)\forall \eta \in g \end{array}$$

#### 5.2.5. The Metric Structure of the Symmetric Dissipative Metriplectic Bracket 

For the Fokker–Planck equation in Hamiltonian systems, Naoki Sato [117,118] observed that $g_{ij}=J_{ik}J_{jk}$ appears in the form inside the dissipative bracket generating the diffusion operator. Then, he deduced that the Poisson operator is linked with the metriplectic dissipative bracket. In the case of dimension two, the covariant version of the tensor is given by a Euclidean metric tensor in the phase space coordinates *(p, q)*:

(96) g=dp⊗dp+dq⊗dq

to be compared with the canonical form of the symplectic 2-form associated with Hamiltonian mechanics:

(97) ω=dp∧dq=dp⊗dp−dq⊗dq

Sato observed that “***the canonical form of the symmetric dissipative part of the metriplectic bracket is identified in terms of a ‘canonical metric tensor’ corresponding to an Euclidean metric tensor on the symplectic leaves foliated by the Casimir invariants***”.

A single generating function $\Phi =\langle \beta ,Q\rangle -S$ is useful to deploy the dynamics by the metriplectic bracket flow:

(98) $$\frac{dF}{dt}=\left\{\left\{F,\Phi \right\}\right\}=\beta^{-1}\left\{F,\Phi \right\}+\left(F,\Phi \right)$$

## 6. Non-Equilibrium Thermodynamic Theory of Dissipative Brackets

Based on non-equilibrium thermodynamics, Baptiste Coquinot has provided new foundations for the dissipative brackets. Poisson brackets and Onsager–Casimir equations both have a connection to Hamiltonian dynamics. For the first time, Coquinot explicitly deduced a generic dissipative bracket from fundamental thermodynamic first principles. The non-equilibrium thermodynamics theories originally presented by Morrison, Grmela and Öttinger and Materassi are covered under this generic bracket. Entropy, a Casimir invariant, serves as the analog to the Hamiltonian in analytical mechanics in the Baptiste Coquinot model, where the non-negativity of the pseudometric ensures the entropy growth associated with the second law of thermodynamics. 

Baptiste Coquinot has used fundamental equations of non-equilibrium thermodynamics describing systems close to thermal equilibrium and irreversible processes. Coquinot has considered σ, the entropy density and $\zeta_{\alpha }$ densities associated with conserved extensive properties with the thermodynamic identity:

(99) $$d\sigma =\sum_{\alpha }X^{\alpha }d\zeta_{\alpha }\;\mathrm{with}\;X^{\alpha }=\frac{\partial \sigma }{\partial \zeta_{\alpha }}$$

All the densities are characterized by the following conservation equations:

(100) $$\frac{\partial \zeta_{\alpha }}{\partial t}+\nabla .J_{\alpha }=0$$

where $\zeta_{\alpha }$ could define a set of dynamical variables and $J_{\alpha }$ an unknown flux associated with $\zeta_{\alpha }$.

Then, the following equation of motion describes the evolution of entropy:

(101) $$\frac{\partial \sigma }{\partial t}+\nabla .J_{T}=\sum_{\alpha }J_{\alpha }.\nabla X^{\alpha }\;\mathrm{with}\;J_{T}=\sum_{\alpha }X^{\alpha }J_{\alpha }$$

where $\nabla X^{\alpha }$ is the affinity associated with the density $\zeta_{\alpha }$ and flux $J_{\alpha }$.

The classical non-equilibrium thermodynamics model assumes a linear response close to the equilibrium:

(102) $$\;J_{\alpha }=\sum_{\beta }L_{\alpha \beta }\nabla X^{\beta }\;,\;\forall \alpha$$

Onsager and Casimir have established contraints on tensor $L=\left[L_{\alpha \beta }\right]$, and more especially, due to physics contraints, that $L=\left[L_{\alpha \beta }\right]$ should be symmetric and positive-definite for entropy growth.

At this step, Baptiste Coquinot has established the dynamics generated with a bracket with respect to the tensor $L=\left[L_{\alpha \beta }\right]$, proving a formal equivalence between the classical out-of-equilibrium thermodynamics and metriplectic dynamical systems. Baptiste Coquinot has shown that the pseudometric nature of the usually empirical dissipative bracket could be deduced from the second law of thermodynamics and Onsager–Casimir relations. 

Considering the phase space with the basis $\left\{\zeta_{\alpha }\right\}$ in which the entropy is geometrically constructed, Baptiste Coquinot has shown how $L=\left[L_{\alpha \beta }\right]$ is related to a bracket on the phase space by rewriting the evolution equations, at a space point x and time t. To establish this relation, Coquinot writes:

(103) $$\begin{array}{ll} \frac{\partial \zeta_{\alpha }(x,t)}{\partial t} & =-\nabla .J_{\alpha }(x,t)=-\nabla .\left[L_{\alpha \beta }(x,t)\nabla \left(\frac{\partial \sigma }{\partial \xi_{\beta }}\right)(x,t)\right]\\ & =-\int_{\Omega }\left[\delta_{\Omega }(x-y)\nabla .\left[L_{\alpha \beta }\left(y,t\right)\nabla \left(\frac{\partial \sigma }{\partial \xi_{\beta }}(y,t)\right)\right]\right]d^{3}y\\ & =\int_{\Omega }\left[\nabla \left(\frac{\partial \zeta_{\alpha }(x,t)}{\partial \zeta_{\gamma }(y,t)}\right)L_{\gamma \beta }\left(y,t\right)\nabla \left(\frac{\partial S}{\partial \xi_{\beta }(y,t)}\right)\right]d^{3}y\;\mathrm{where}\;S=\int_{\Omega }\sigma \end{array}$$

Coquinot then deduces the equation on the dynamics of Entropy:

(104) $$\begin{array}{ll} \frac{\partial \sigma (x,t)}{\partial t} & =-\nabla .J_{T}(x,t)+J_{\alpha }.\nabla \left(\frac{\partial \sigma }{\partial \zeta_{\alpha }}\right)(x,t)\\ & =-\nabla .\left[\frac{\partial \sigma }{\partial \zeta_{\alpha }}(x,t)L_{\alpha \beta }(x,t)\nabla \left(\frac{\partial \sigma }{\partial \zeta_{\alpha }}\right)(x,t)\right]+\nabla \left(\frac{\partial \sigma }{\partial \zeta_{\alpha }}\right)(x,t)L_{\alpha \beta }(x,t)\nabla \left(\frac{\partial \sigma }{\partial \zeta_{\alpha }}\right)(x,t)\\ & =-\frac{\partial \sigma }{\partial \zeta_{\alpha }}(x,t)\nabla .\left[L_{\alpha \beta }(x,t)\nabla \left(\frac{\partial \sigma }{\partial \zeta_{\alpha }}\right)(x,t)\right]\\ & =\int_{\Omega }\left[\nabla \left(\frac{\partial \sigma }{\partial \zeta_{\alpha }}(y,t)\delta_{\Omega }(x-y)\right)L_{\alpha \beta }\left(y,t\right)\nabla \left(\frac{\partial \sigma }{\partial \zeta_{\beta }}(y,t)\right)\right]d^{3}y\\ & =\int_{\Omega }\left[\nabla \left(\frac{\partial \sigma (x,t)}{\partial \zeta_{\alpha }(y,t)}\right)L_{\alpha \beta }\left(y,t\right)\nabla \left(\frac{\partial S(t)}{\partial \zeta_{\beta }(y,t)}\right)\right]d^{3}y \end{array}$$

Coquinot deduced that the dynamics of out-of-equilibrium thermodynamics on the phase space can be expressed with a symmetric bracket for any two functionals f and g:

(105) $$\left(f,g\right)=\frac{1}{ϒ}\int_{\Omega }\nabla \left(\frac{\partial f}{\partial \zeta_{a}(y)}\right)L_{\alpha \beta }\nabla \left(\frac{\partial g}{\partial \zeta_{\beta }(y)}\right)d^{3}y$$

Coquinot has observed that this equation is a pure geometric object, independent of the basis $\left\{\zeta_{\alpha }\right\}$, where the functional derivatives can be seen as functional gradients, and both functional gradients are contracted thanks to the pseudometric $\left[L_{\alpha \beta }\right]$ and where the bracket is symmetric thanks to the Onsager–Casimir relations. 

As previously, it has been demonstrated that:

(106) $$\frac{\partial \sigma (x,t)}{\partial t}=\int_{\Omega }\left[\nabla \left(\frac{\partial \sigma (x,t)}{\partial \zeta_{\alpha }(y,t)}\right)L_{\alpha \beta }\left(y,t\right)\nabla \left(\frac{\partial S(t)}{\partial \zeta_{\beta }(y,t)}\right)\right]d^{3}y=ϒ\left(\sigma ,S\right)$$

Coquinot writes the evolution of any functional f as:

(107) $$\frac{df}{dt}=ϒ\left(f,S\right)$$

To include the first law of thermodynamics, Coquinot proposes ε as a basic variable in the thermodynamic framework ($\frac{\partial H}{\partial \varepsilon }$ is then unity and the other elements of the basis are independent of ε) and obtains, for any functional f, the following:

(108) $$\left(f,H\right)=0$$

By construction, the non-equilibrium thermodynamics preserves $\int_{\Omega }\zeta_{\alpha }$ for any α, and ε is chosen as one of the $\zeta_{\alpha }$ values, and the dissipative bracket formulates the first law of thermodynamics. With bilinearity, symmetry and degeneracy properties, a metriplectic dynamical system results from coupling such a bracket with the accompanying noncanonical Poisson bracket.

The following is a quote from Baptiste Coquinot: “Our construction above shows that the dissipative brackets are entirely natural for non-equilibrium thermodynamics, just as the Poisson brackets are natural for Hamiltonian dynamics. Above, we explicitly deduced a generic dissipative bracket from fundamental thermodynamic first principles, presumably for the first time. This general bracket includes non-equilibrium thermodynamic theories that resemble fluids, such as those initially proposed by Morrison and later by others (Grmela & Öttinger, Edwards, Materassi & Tassi)”. This Coquinot development provides a non-equilibrium thermodynamics point of view to found dissipative bracket theory. It does so by first taking into account variables for building the bracket of the metriplectic flow and then by proposing a methodical approach to derive brackets for dissipation. The Onsager–Casimir reciprocity relations describe time-reversal invariance at the microscopic scale for the relaxation of quantities at the macroscopic scale close to thermodynamic equilibrium in the linear regime and link time reversibility at the microscopic scale with a symmetry property of corresponding evolution equations at the macroscopic scale. Through this equivalence, Baptiste Coquinot establishes a formal equivalence between a metriplectic dynamical systems subclass and classical out-of-equilibrium thermodynamics, demonstrating that the dissipative bracket pseudometric nature, which is typically a hypothesis, is the exact transcription of the second principle of thermodynamics and Onsager–Casimir relations. By deriving a generic dissipative bracket from fundamental thermodynamic first principles, Coquinot’s design demonstrates that brackets for dissipation are canonical for non-equilibrium thermodynamics, just as Hamiltonian dynamics Poisson brackets are canonical. According to Baptiste Coquinot, entropy, a Casimir invariant, plays a similar role to the Hamiltonian in analytical mechanics. The pseudometric’s non-negativity assures that entropy continues to increase in accordance with the second law of thermodynamics. According to Baptiste Coquinot, the natural variables that make up the basis of the phase space are different from one another from both a Hamiltonian and a thermodynamic standpoint, but one can change the variables by using the thermodynamic identity to obtain a bracket in any full set of phase space variables. The first law of thermodynamics is well formulated in the Coquinot dissipative bracket, with respect to all the characteristics. The linkage of such a bracket with the corresponding noncanonical Poisson bracket was first noticed by Baptiste Coquinot.

## 7. Dirac’s Theory of Constrained Hamiltonian Systems: Dissipative Bracket

For Lagrangian systems with degenerate Lagrangians, Paul Dirac introduced the generalized Hamiltonian dynamics in 1950. For the Dirac model, the system phase space is endowed with two Poisson brackets: the Poisson bracket of the standard case deduced from its symplectic structure, and the Dirac bracket.

The Dirac restricted bracket could be used to infer the transverse Poisson structure. Using Dirac’s constraint bracket formula, Oh [119] defined the requirements in 1986 for the transverse Poisson structure to a coadjoint orbit to be at most quadratic. Let $\left(M,\left\{,\right\}\right)$ be a Poisson manifold, x a point in M, ℘ be the symplectic leaf through x, N be a transverse submanifold to ℘ at x, U be the neighborhood of x and y∈U. Consider functions $\psi_{1},\ldots ,\psi_{2n}$ such that $N=\left\{y\in U/\psi_{1}(y)=0,\ldots ,\psi_{2n}(y)=0\right\}$ and denote by C the non-singular matrix such that $C_{ij}(y)=\left\{\psi_{i},\psi_{j}\right\}(y)$. Then the transverse Poisson structure to ℘ is given by:

(109) $${\left\{f,h\right\}}_{N}=\left\{\tilde{f},\tilde{h}\right\}-\sum_{i,j=1}^{2n}\left\{\tilde{f},\psi_{i}\right\}{\left(C^{-1}\right)}_{ij}\left\{\psi_{j},\tilde{h}\right\}$$

where $f,h\in C^{\infty }\left(N\right)$ and $\tilde{f},\tilde{h}$ are arbitrary extensions to U of f,h. 

Dirac proved the Jacobi identity for this bracket, relying heavily on the invertibility of C, and as a consequence of the constraints are Casimir invariants, ${\left\{\psi_{i},h\right\}}_{N}=0\;,\;\forall h$.

Michel Saint-Germain used natural identifications typical of the Lie–Poisson scenario to derive from Dirac’s constraint bracket formula, a formula for computing the transverse Poisson structure for a coadjoint orbit. Damianou then hypothesized that the transverse Poisson structure to any coadjoint orbit is polynomial for a semisimple Lie algebra. Cushman and Roberts’ research has then demonstrated this conjecture.

Cristel Chandre has more recently examined Dirac’s theory of constrained Hamiltonian systems, demonstrating that the identity of Jacobi arises from the constraints considered as Casimir invariants, for any reversibility of the Poisson bracket matrix. The validity of the identity given by Jacobi is guaranteed throughout phase space, not simply on the surface bounded by the restrictions, according to Cristel Chandre’s demonstration.

Cristel Chandre has considered a finite-dimensional Hamiltonian system with variables $z=\left(z_{1},\ldots ,z_{n}\right)$, given by a Hamiltonian H(z), and a Poisson bracket:

(110) $$\left\{f,h\right\}=\frac{\partial f}{\partial z}J(z)\frac{\partial h}{\partial z}$$

J(z), the Poisson matrix, is associated with a bracket that is antisymmetric with the Jacobi identity in addition to the Leibnitz rule and the bilinearity, ensured by the bracket form.

m < n − 2 constraints are imposed from the variable *z*; $\psi_{i}(z)=0$ for *i* = 1, …, m. Dirac brackets given by an antisymmetric matrix D are not in the general Poisson bracket and the inverse of C. The matrix associated with the Dirac bracket is given by:

(111) $$J_{N}=J-J{Κ}^{+}DKJ$$

where the matrix K has elements $K_{li}=\frac{\partial \psi_{l}}{\partial z_{i}}$. The matrix D is defined with the property that the constraints are Casimir invariants, leading to the following condition for D:

(112) $$JK^{+}\left(I-DC\right)=0\;\mathrm{with}\;C=KJK^{+}$$

In this way, it has been demonstrated how Jacobi identity can be attained.

## 8. Transverse Poisson Structure for Dissipative Heat Equation Introduced by Herve Sabourin

By restricting consideration to the case of nilpotent orbits, Hervé Sabourin has examined the coadjoint orbit transverse Poisson structure in a complex Lie algebra, considered as semisimple. Hervé Sabourin demonstrated that a determinantal formula based on Chevalley’s restriction of the invariants on the slice may adequately characterize the transverse Poisson structure for nilpotent orbits with a subregular property. The transverse Poisson structure is transformed into a three-dimensional Poisson bracket based on the Slodowy slice model.

When M is the dual Lie algebra $g^{*}$ of a complex Lie algebra g, it is equipped with Lie–Poisson structure in standard form. The Killing form $\langle .,.\rangle$ of g identifies g with its dual $g^{*}$, and a Poisson structure on g is given for functions f and g on g at x∈g by $\left\{f,g\right\}(x)=\langle x,\left[df(x),dg(x)\right]\rangle$ where df(x) and dg(x) are elements of $g≅g^{*}≅T_{x}^{*}g$. By means of the ad-invariant Killing form, the adjoint orbits G.x of G are identified with the coadjoint orbits G.μ, by the isomorphism $g≅g^{*}$. The symplectic leaf through $\mu \in g^{*}$ is the coadjoint orbit G.μ of the adjoint Lie group G of g.

A transverse slice to G.x is given by choosing any complement n to the centralizer $g(x)=\left\{y\in g/\left[x,y\right]=0\right\}$ of x in g and by taking N as the affine subspace $x+n^{⊥}$ of $g^{*}$, where ⊥ is the orthogonal complement with respect to the Killing form. Using the property that $g{\left(x\right)}^{⊥}=ad_{g}^{*}x$, we have the following decomposition: $T_{x}\left(g^{*}\right)=T_{x}\left(G.x\right)⊕T_{x}\left(N\right)$, where N is a transverse slice to G.x at x. Sabourin has given an explicit formula for the Poisson structure ${\left\{.,.\right\}}_{N}$ transverse to G.x. Let $\left(Z_{1},\cdots ,Z_{k}\right)$ be a basis for g(x), and let $\left(X_{1},\cdots ,X_{2r}\right)$ be a basis for n, where $2r=\dim\left(G.x\right)$ is the rank of the Poisson structure at x. These bases lead to linear coordinates $q_{1},\cdots ,q_{k+2r}$ on g, centered at x, defined by $q_{i}(y)=\langle y-x,Z_{i}\rangle$ for i=1,⋯,k with $dq_{i}(y)=Z_{i}$, and $q_{k+i}(y)=\langle y-x,X_{i}\rangle$ for i=1,⋯,2r with $dq_{k+i}(y)=X_{i}$. The Poisson matrix $\left\{.,.\right\}$ at y∈g is given by:

(113) $${\left(\left\{q_{i},q_{j}\right\}(y)\right)}_{1\leq i,j\leq k+2r}=\left(\begin{array}{cc} A(y) & B(y)\\ -B{\left(y\right)}^{T} & C(y) \end{array}\right)\;\mathrm{where}\;\left\{\begin{array}{l} A_{i,j}(y)=\langle y,\left[Z_{i},Z_{j}\right]\rangle \;,\;\mathrm{for}\;1\leq i,j\leq k\\ B_{i,m}(y)=\langle y,\left[Z_{i},X_{m}\right]\rangle \;,\;\mathrm{for}\;1\leq i\leq k,\;1\leq m\leq 2r\\ C_{l,m}(y)=\langle y,\left[X_{i},X_{m}\right]\rangle \;,\;\mathrm{for}\;1\leq l,m\leq 2r \end{array}\right.$$

C(x) is a skew-symmetric invertible matrix, and so C(y) is invertible for y in a neighborhood of x in g, and hence for y in a neighborhood V of x in N. Then, the Poisson matrix of ${\left\{.,.\right\}}_{N}$ at n∈V in the coordinates $q_{1},\cdots ,q_{k}$ (restricted to V) is given by Dirac reduction:

(114) $$\Lambda_{N}=A(n)+B(c)C{\left(n\right)}^{-1}B{\left(n\right)}^{T}$$

For the case of semisimple Lie algebra, Sabourin has used the Jordan–Chevalley decomposition theorem, where x could be decomposed in x=s+e, where s is semisimple, e is nilpotent and $\left[s,e\right]=0$, with following relation between centralizers: g(x)=g(s)∩g(e). We have the vector space decomposition $g=g(s)⊕n_{s}\;\mathrm{where}\;n_{s}=g{\left(s\right)}^{⊥}$, and $n_{s}$ is g(s) invariant such that $\left[g(s),n_{s}\right]\subset n_{s}$ (observing that $\langle g(s),\left[g(s),n_{s}\right]\rangle =\langle \left[g(s),g(s)\right],n_{s}\rangle \subset \langle g(s),n_{s}\rangle =\left\{0\right\}$). Sabourin has then considered the following decomposition $g=g(x)⊕n_{e}⊕n_{s}$ where $n_{e}$ is any complement of g(x) in g(s). Then taking $n=n_{e}⊕n_{s}$, Sabourin has denoted $N_{x}=x+n^{⊥}$ and has proved that if $n\in N_{x}$, such that n=g(s), then $\langle n,\left[g(s),n_{s}\right]\rangle \subset \langle g(s),n_{s}\rangle =\left\{0\right\}$ and in particular $\langle n,\left[g(x),n_{s}\right]\rangle =\left\{0\right\}\;\mathrm{and}\;\langle n,\left[n_{e},n_{s}\right]\rangle =\left\{0\right\}$. Considering the basis vectors $X_{1},\cdots ,X_{2r}$ of n such that $X_{1},\cdots ,X_{2p}\in n_{e}$ and $X_{2p+1},\cdots ,X_{2r}\in n_{s}$ and as $\langle n,\left[g(x),n_{s}\right]\rangle =\left\{0\right\}\;\mathrm{and}\;\langle n,\left[n_{e},n_{s}\right]\rangle =\left\{0\right\}$, then the Poisson matrix takes at $n\in N_{x}$ the form:

(115) $$\Lambda (n)=\left(\begin{array}{ccc} A(n) & B_{e}(n) & 0\\ -B_{e}{\left(n\right)}^{T} & C_{e}(n) & 0\\ 0 & 0 & C_{s}(n) \end{array}\right)\;\mathrm{where}\;\left\{\begin{array}{l} A_{i,j}(n)=\langle n,\left[Z_{i},Z_{j}\right]\rangle \;,\;\mathrm{for}\;1\leq i,j\leq k\\ B_{e;}{}_{i,m}(n)=\langle n,\left[Z_{i},X_{m}\right]\rangle \;,\;\mathrm{for}\;1\leq i\leq k,\;1\leq m\leq 2p\\ C_{e;}{}_{l,m}(y)=\langle n,\left[X_{i},X_{m}\right]\rangle \;,\;\mathrm{for}\;1\leq l,m\leq 2p\\ C_{s;}{}_{l,m}(y)=\langle n,\left[X_{i},X_{m}\right]\rangle \;,\;\mathrm{for}\;2p+1\leq l\;,\;m\leq 2r \end{array}\right.$$

Sabourin has deduced from it that the Poisson matrix of the transverse Poisson structure on $N_{x}$ is given by:

(116) $$\Lambda_{N_{x}}(n)=A(n)+B_{e}(c)C_{e}{\left(n\right)}^{-1}B_{e}{\left(n\right)}^{T}\;(*)\;$$

Sabourin has also proved that:

(117) $$\Lambda_{N}=A(n)+B_{e}(c)C_{e}{\left(n\right)}^{-1}B_{e}{\left(n\right)}^{T}\;\mathrm{where}\;n\in N$$

yielding formally the same formula, except that it is evaluated at points n of N rather than at points of $N_{x}$, and providing the following proposition:

**Sabourin** **Proposition:** *Let*x∈g*be any element*, G.x*its adjoint orbit, and*x=s+e*its Jordan–Chevalley decomposition. Given any complement*$n_{e}$*of*g(x)*in*g(s)*and putting*$n=n_{s}⊕n_{e}$*, where*$n_{s}=g{\left(s\right)}^{⊥}$*, the parallel affine spaces*$N_{x}=x+n^{⊥}$*and*$N=e+n^{⊥}$*are respectively transverse slices to the adjoint orbit*G.x*in*g*and to the nilpotent orbit*G(s).e*in*g(s). The Poisson structure on both transverse slices has the same Poisson matrix, namely that of (*), in the same affine coordinates restricted to the corresponding transverse slice.

In short, given by Sabourin, the transverse Poisson structure to any adjoint orbit G.x of a semisimple (or reductive) Lie algebra g is essentially determined by the transverse Poisson structure of the underlying nilpotent orbit G(s).e defined by the Jordan–Chevalley decomposition x=s+e. Sabourin has also proved that the transverse Poisson structure on $N=e+n^{⊥}$ is a polynomial Poisson structure that is quasihomogeneous of degree −2.

More explicitly, Sabourin has explicitly described the subregular orbit adjoint transverse Poisson structure $O_{sr}\subset g$, where g is a semisimple Lie algebra. The generic rank of the transverse slice N adjoint transverse Poisson structure is two, and dim(N)−2 independent Casimirs are known; Sabourin has derived that the adjoint transverse Poisson structure is the determinantal structure determined by these Casimirs, up to multiplication by a constant, based on the theory of simple singularities introduced by Brieskorn and Slodowy slice model.

The following result is given by Sabourin on invariant functions and Casimirs. Let $O_{sr}=G.e$ be a subregular orbit in the semisimple Lie algebra g. Let $\left(h,e,f\right)$ be the corresponding canonical sl2-triple, and consider the transverse slice $N=e+n^{⊥}$ to G.e, where n is an $ad_{h}$-invariant complement to g(e). Let $S{\left(g^{*}\right)}^{G}$ be the algebra of ad-invariant polynomial functions on g, then $S{\left(g^{*}\right)}^{G}$ is a polynomial algebra generated by l homogeneous polynomials $\left(G_{1},\cdots ,G_{l}\right)$ deduced from a classical theorem due to Chevalley. These functions are Casimirs of the Lie–Poisson structure on g, since ad-invariance of $G_{i}$ implies that $\left[x,dG_{i}(x)\right]=0$, and hence the Lie–Poisson bracket is:

$\left\{F,G_{i}\right\}(x)=\langle x,\left[dF(x),dG_{i}(x)\right]\rangle =-\langle \left[x,dG_{i}(x)\right],dF(x)\rangle =0$ for any function F on g. 

Sabourin has observed that if we denote by $\chi_{i}$ the restriction of $G_{i}$ to the transverse slice N, then it follows that these functions are Casimirs of the adjoint transverse Poisson structure. 

In the Lie–Poisson case, M. Saint-Germain has proved in his PhD the rationality of the transverse Poisson structure. This result has been completed by P. Damianou for coadjoint orbits in a semisimple Lie algebra, and by Hervé Sabourin in 2005.

Via the Jordan–Chevalley decomposition of x∈g, Hervé Sabourin has studied the transverse Poisson structure to any adjoint orbit G.x and has proved that it can be reduced to the case of an adjoint nilpotent orbit. 

As the transverse structure to the regular nilpotent orbit $O_{reg}$ of g is always trivial, Hervé Sabourin has considered the case of the subregular nilpotent orbit $O_{sr}$ of g with dimension of $O_{sr}$ two less than the dimension of the regular orbit, so that the transverse Poisson structure has rank 2. Sabourin has proved that we could replace, for the transverse Poisson structure, the complicated Dirac constraints with a simple determinantal formula:

(118) $${\left\{f,g\right\}}_{\det}=\det\left(\nabla f,\nabla g,\nabla \chi_{1},\ldots ,\nabla \chi_{l}\right)=\frac{df\wedge dg\wedge d\chi_{1}\wedge \ldots \wedge d\chi_{l}}{dq_{1}\wedge dq_{2}\wedge \ldots \wedge dq_{l+2}}$$

where $\chi_{1},\ldots ,\chi_{l}$ are independent polynomial Casimir functions with $\chi_{i}$ the restriction of the *i*-th Chevalley invariant $G_{i}$ to the slice N, and $q_{1},\ldots ,q_{l+2}$ are linear coordinates on N. 

Sabourin has proved the following theorem:

**Sabourin** **Theorem:** *Let* $O_{sr}$ *be the subregular nilpotent adjoint orbit of a complex semisimple Lie algebra* g *, and let* $\left(h,e,f\right)$ *be the canonical triple associated with* $O_{sr}$ *. Let* $N=e+n^{⊥}$ *be a slice transverse to* $O_{sr}$ *, where* n *is an* $ad_{h}$ *-invariant complementary subspace to* g(e) *. Let* ${\left\{.,.\right\}}_{N}$ *and* ${\left\{.,.\right\}}_{\det}$ *denote respectively the adjoint transverse Poisson structure and the determinantal structure on* N *. Then* ${\left\{.,.\right\}}_{N}=c{\left\{.,.\right\}}_{\det}$ *for some* $c\in {\mathbb{C}}^{*}$

From this determinantal formula, Sabourin has deduced that the Poisson matrix of the transverse Poisson on N takes, in suitable coordinates, the block form:

(119) $$\tilde{\Lambda }=\left(\begin{array}{cc} 0 & 0\\ 0 & \Omega \end{array}\right)\;\mathrm{where}\;\Omega =c^{\prime }\left(\begin{array}{ccc} 0 & \frac{\partial F}{\partial q_{l+2}} & -\frac{\partial F}{\partial q_{l+1}}\\ -\frac{\partial F}{\partial q_{l+2}} & 0 & \frac{\partial F}{\partial q_{l}}\\ \frac{\partial F}{\partial q_{l+1}} & -\frac{\partial F}{\partial q_{l}} & 0 \end{array}\right)$$

where F is the polynomial $F\left(u_{1},\ldots ,u_{l-1},q_{l},q_{l+1},q_{l+2}\right)$ with $u_{1},\ldots ,u_{l-1}$ being the deformation parameters, given by Casimir for the Poisson structure on N, of simple singularity of the singular surface N∩ℵ, where ℵ is the nilpotent cone of g.

## 9. Application for SL(2,R) and SU(1,1)

For SU(1,1)/U(1) group acting transitively on Poincaré unit disk, we will introduce the moment map. Considering the Lie group:

(120) $$SU(1,1)=\left\{\left(\begin{array}{cc} a & b\\ b^{*} & a^{*} \end{array}\right)=\left(\begin{array}{cc} 1 & ba^{*-1}\\ 0 & 1 \end{array}\right)\left(\begin{array}{cc} a^{*-1} & 0\\ 0 & a^{*} \end{array}\right)\left(\begin{array}{cc} 1 & 0\\ a^{*-1}b^{*} & 1 \end{array}\right)/a,b\in R,\;{\left|a\right|}^{2}-{\left|b\right|}^{2}=1\right\}$$

and its Lie algebra given by elements:

(121) $$su(1,1)=\left\{\left(\begin{array}{cc} ir & \eta \\ \eta^{*} & -ir \end{array}\right)/r\in R,\eta \in C\right\}$$

a basis for this Lie algebra su(1,1) is $\left(u_{1},u_{2},u_{3}\right)\in g$ with

(122) $$u_{1}=\frac{i}{2}\left(\begin{array}{cc} 1 & 0\\ 0 & -1 \end{array}\right),\;u_{2}=-\frac{1}{2}\left(\begin{array}{cc} 0 & 1\\ 1 & 0 \end{array}\right)\;\mathrm{and}\;u_{3}=\frac{1}{2}\left(\begin{array}{cc} 0 & -i\\ i & 0 \end{array}\right)$$

with $\left[u_{1},u_{3}\right]=-u_{2},\left[u_{1},u_{2}\right]=u_{3},\left[u_{2},u_{3}\right]=-u_{1}$. The Harish–Chandra embedding is given by $\varphi \left(gx_{0}\right)=\zeta =ba^{*-1}$. From ${\left|a\right|}^{2}-{\left|b\right|}^{2}=1$, one has $\left|\zeta \right|<1$. Conversely, for any $\left|\zeta \right|<1$, taking any a∈C such that $\left|a\right|={\left(1-{\left|a\right|}^{2}\right)}^{-1/2}$ and putting $b=\zeta a^{*}$, one obtains g∈G for which $\varphi \left(gx_{0}\right)=\zeta$. The domain D=φ(M) is the unit disk $D=\left\{\zeta \in C/\left|\zeta \right|<1\right\}$.

The compact subgroup is generated by $u_{1}$, while $u_{2}$ and $u_{3}$ generate a hyperbolic subgroup. The dual space of the Lie algebra is given by:

(123) $$su{\left(1,1\right)}^{*}=\left\{\left(\begin{array}{cc} z & x+iy\\ -x+iy & -z \end{array}\right)/x,y,z\in R\right\}$$

with the basis $\left(u_{1}^{*},u_{2}^{*},u_{3}^{*}\right)\in g^{*}$:

(124) $$u_{1}^{*}=\left(\begin{array}{cc} 1 & 0\\ 0 & -1 \end{array}\right),u_{2}^{*}=\left(\begin{array}{cc} 0 & i\\ i & 0 \end{array}\right)\;\mathrm{and}\;u_{3}^{*}=\left(\begin{array}{cc} 0 & 1\\ -1 & 0 \end{array}\right)$$

Let us consider $D=\left\{z\in C/\left|z\right|<1\right\}$ as the open unit disk of Poincaré. For each ρ>0, the pair $\left(D,\omega_{\rho }\right)$ is a symplectic homogeneous manifold with $\omega_{\rho }=2i\rho \frac{dz\wedge dz^{*}}{{\left(1-{\left|z\right|}^{2}\right)}^{2}}$, where $\omega_{\rho }$ is invariant under the action:

(125) $$\begin{array}{l} SU\left(1,1\right)\times D\to D\\ \left(g,z\right)\mapsto g.z=\frac{az+b}{b^{*}z+a^{*}} \end{array}$$

This action is transitive and is globally and strongly Hamiltonian. Its generators are the Hamiltonian vector fields associated with the functions:

(126) $$J_{1}(z,z^{*})=\rho \frac{1+{\left|z\right|}^{2}}{1-{\left|z\right|}^{2}},J_{2}(z,z^{*})=\frac{\rho }{i}\frac{z-z^{*}}{1-{\left|z\right|}^{2}},\;J_{3}(z,z^{*})=-\rho \frac{z+z^{*}}{1-{\left|z\right|}^{2}}$$

The associated moment map $J:D\to su^{*}(1,1)$ defined by $J(z).u_{i}=J_{i}(z,z^{*})$, maps D into a coadjoint orbit in $su^{*}(1,1)$. Then, we can write the moment map as a matrix element of $su^{*}(1,1)$:

(127) $$J(z)=J_{1}\left(z,z^{*}\right)u_{1}^{*}+J_{2}\left(z,z^{*}\right)u_{2}^{*}+J_{3}\left(z,z^{*}\right)u_{3}^{*}=\rho \left(\begin{array}{cc} \frac{1+{\left|z\right|}^{2}}{1-{\left|z\right|}^{2}} & -2\frac{z^{*}}{1-{\left|z\right|}^{2}}\\ 2\frac{z}{1-{\left|z\right|}^{2}} & -\frac{1+{\left|z\right|}^{2}}{1-{\left|z\right|}^{2}} \end{array}\right)\in g^{*}$$

The moment map J is a diffeomorphism of D onto one sheet of the two-sheeted hyperboloid in $su^{*}(1,1)$, given by $J_{1}^{2}-J_{2}^{2}-J_{3}^{2}=\rho^{2}\;,\;J_{1}\geq \rho \;\mathrm{with}$$J_{1}u_{1}^{*}+J_{2}u_{2}^{*}+J_{3}u_{3}^{*}\in su^{*}(1,1)$. We note $O_{\rho }^{+}$, the coadjoint orbit $Ad_{SU(1,1)}^{*}$ of $SU\left(1,1\right)$, given, for the two-sheeted hyperboloid, by the upper sheet. The KKS orbit method associates each of these coadjoint orbits with a representation of the discrete series of $SU\left(1,1\right)$, provided that ρ is a half-integer greater than or equal to 1 ($\rho =\frac{k}{2},k\in N\;\mathrm{and}\;\rho \geq 1$). When explicitly executing the KKS construction, the representation Hilbert spaces $H_{\rho }$ are realized as closed reproducing kernel subspaces of $L^{2}\left(D,\omega_{\rho }\right)$.The KKS orbit method shows that each coadjoint orbit of a connected Lie group is associated with a unitary irreducible representation of G acting in a Hilbert space *H*.

Since the unit disk is Kählerian, it is symplectic and so can be given a phase space structure and interpretation. This Poisson bracket could be written in terms of the Poincaré disk coordinates as:

(128) $$\left\{f,g\right\}=\frac{{\left(1-{\left|z\right|}^{2}\right)}^{2}}{2i}\left(\frac{\partial f}{\partial z}\frac{\partial g}{\partial z^{*}}-\frac{\partial f}{\partial z^{*}}\frac{\partial g}{\partial z}\right)$$

It is possible to define new coordinates $\left(q,p\right)$ that are canonical in the sense that:

(129) $$\left\{f,g\right\}=\left(\frac{\partial f}{\partial q}\frac{\partial g}{\partial p}-\frac{\partial f}{\partial p}\frac{\partial g}{\partial q}\right)$$

with coordinates given by:

(130) $$\frac{q+ip}{2}=\frac{z}{\sqrt{1-{\left|z\right|}^{2}}}$$

The metriplectic equation is then given by:

(131) $$\frac{\partial f}{\partial t}=\left\{f,H\right\}+\left(f,H\right)=\frac{{\left(1-{\left|z\right|}^{2}\right)}^{2}}{2i}\left(\frac{\partial f}{\partial z}\frac{\partial H}{\partial z^{*}}-\frac{\partial f}{\partial z^{*}}\frac{\partial H}{\partial z}\right)+\left(f,H\right)$$

**Figure 4:**
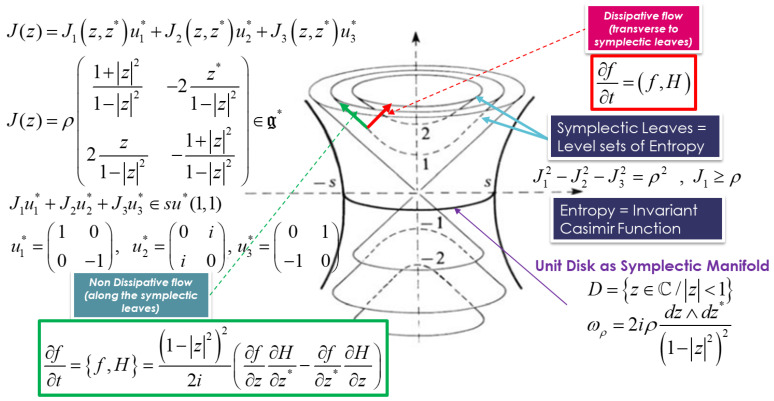
Souriau Model for SU(1,1) Lie Group and Poincaré Unit Disk (Metriplectic Model).

These equations for SU(1,1) Lie group are illustrated in Figure 4.

## 10. Lindblad Equation and Metriplectic Model

We shall explore the equation introduced by Lindblad, the dissipative Hamiltonian Liouville equation of the quantum density matrix, to illustrate earlier models. To demonstrate that the equation introduced by Lindblad can be written as a damped Hamiltonian system and the GENERIC (General Equation of Non-Equilibrium Reversible and Irreversible Coupling) model, which was developed by Grmela and Öttinger, we will use the study performed by Markus Mittnenzweig and Alexander Mielke. They have expressed the dissipative portion of the Lindblad operator as a gradient of relative entropy, demonstrating how the maximum entropy principle can lead to equilibria.

As noted by H.C. Öttinger, after formulating the thermodynamically consistent nonlinear master equation, one can search for unique circumstances in which precise or approximative linear master equations can be generated. H. Graber has conducted this, and he has come to the conclusion that the master equations that result are not of the well-known Lindblad form. The thermodynamic quantum master equation’s nonlinearity is its most notable characteristic. The linear Liouville and Schrödinger equations, which describe reversible classical and quantum systems, as well as the Fokker–Planck equations, which describe irreversible classical systems, are fundamentally different from this. The nonlinearity is brought on by the interaction of quantum mechanics and irreversible thermodynamics. Even the harmonic oscillator’s master equation exhibits severe nonlinearity. The well-known master equations of the Lindblad type ignore this underlying nonlinearity. The thermodynamic master equations’ solutions, like those for the linear Lindblad equations, always remain in the physical domain, which is known to be a subtle problem for nonlinear equations. H.C. Öttinger’s work demonstrates how nonlinearity can be handled for the two-level system. Since noncommutativity results in quantum nonlinearity, Öttinger’s master equation cannot be of the standard Lindblad type. The symmetric anticommutator provides the most natural linearization of the GENERIC master equation. Linearizations, however, ruin the thermodynamic structure and are hence not advised. The linearized equation is then of the Lindblad form, where the Hamiltonian must be redefined and a Lindblad operator with real and imaginary parts introduced. The thermodynamic arguments prove that the quantum master equation should be extremely nonlinear. The thermodynamic structure leads to a clear formulation of a nonlinear quantum master equation. The linearized GENERIC quantum master equation developed by H.C. Öttinger is generated following approximation in the second term that is used to reconstruct the standard Lindblad master equation. The validity range is lowered by the thermodynamic nonlinearity, which naturally results in canonical equilibrium solutions.

The dissipative evolution equation of quantum systems governed by Hamiltonian and dissipative effects is given on the density matrix $\rho =\rho^{*}\geq 0$:

(132) $$\begin{array}{l} \frac{\partial \rho }{\partial t}=\frac{1}{\hbar }\left[\rho ,H\right]+ℑ\rho \\ \mathrm{with}\;\left[\rho ,H\right]=\rho H-H\rho \\ \mathrm{and}\;ℑ\rho =\sum_{n,m=1}^{N^{2}-1}a_{n,m}\left(\left[Q_{n},\rho Q_{m}^{*}\right]+\left[Q_{n}\rho ,Q_{m}^{*}\right]\right)\;\mathrm{with}\;\left\{\begin{array}{l} Q_{n}:\;\mathrm{arbitrary}\;\mathrm{operators}\\ a_{n,m}:\;\mathrm{Hermitian}\;\mathrm{positive}\;\mathrm{semi}-\mathrm{definite}\;\mathrm{matrix} \end{array}\right. \end{array}$$

The entropy production is convex and positive, and H. Spohn has demonstrated that it is a gauge of the semigroup’s dissipativity. Additionally, he has noted that the relative entropy decays along solutions and is a Liapunov function:

(133) $$\begin{array}{l} F\left(\rho \right)=Tr\left(\rho \left(\log\rho -\log{\hat{\rho }}_{\beta }\right)\right)=Tr\left(\rho \left(\log\rho +\beta H\right)\right)+\log Z_{\beta }\\ \mathrm{with}\;{\hat{\rho }}_{\beta }=\frac{1}{Z_{\beta }}e^{-\beta H}\;\mathrm{and}\;Z_{\beta }=Tr\left(e^{-\beta H}\right) \end{array}$$

The dissipative evolution equation can be expressed as a damped Hamiltonian system, as demonstrated by Markus Mittnenzweig and Alexander Mielke:

(134) $$\begin{array}{l} \frac{\partial \rho }{\partial t}=\left(J\left(\rho \right)-K\left(\rho \right)\right)DF\left(\rho \right)\\ \mathrm{where}\\ J\left(\rho \right):\xi \mapsto \frac{i}{\beta }\left[\rho ,\xi \right]\;\mathrm{generating}\;\mathrm{Poisson}\;\mathrm{Bracket}\;\mathrm{with}\;J\left(\rho \right)=-J{\left(\rho \right)}^{*}\\ K\left(\rho \right)=K{\left(\rho \right)}^{*}\;\mathrm{and}\;\langle \xi ,K\left(\rho \right)\xi \rangle \geq 0\;\mathrm{purely}\;\mathrm{dissipative}\\ \langle DF\left(\rho \right),v\rangle =\underset{h\to 0}{Lim}\frac{1}{h}\left(F\left(\rho +hv\right)-F\left(\rho \right)\right)\\ \mathrm{with}\;{\hat{\rho }}_{\beta }=\frac{1}{Z_{\beta }}e^{-\beta H}\;\mathrm{and}\;Z_{\beta }=Tr\left(e^{-\beta H}\right) \end{array}$$

The evolution of the quantum mechanical system can be coupled to more macroscopic dissipative components z and thus with the total state $q=\left(\rho ,z\right)$. They have considered the GENERIC model for isothermal systems with fixed temperature $T^{*}>0$, with the free energy $F\left(q\right)=E\left(q\right)-T^{*}S\left(q\right)$. The associated structure is the following damped Hamiltonian system:

(135) $$\begin{array}{l} \frac{\partial q}{\partial t}=\left(J\left(q\right)-\beta^{*}K\left(q\right)\right)DF\left(q\right)\;\mathrm{where}\;J(q)DS(q)=0\;\mathrm{and}\;K(q)DE(q)=0\\ \mathrm{with}\;\beta^{*}=\frac{1}{T^{*}}\;,\;E\left(\rho ,z\right)=Tr\left(\rho H\right)+E(z)\;,\;S\left(\rho ,z\right)=-k_{B}Tr\left(\rho \log\rho \right)+S(z) \end{array}$$

They proved that equilibria can be obtained by the maximum entropy principle. 

For the Poisson structure, the variable z is totally dissipative, which means that J(q) has a block structure in the following form:

(136) $$J(\rho ,z)=\left[\begin{array}{cc} \frac{i}{\hbar }\left[\rho ,.\right] & 0\\ 0 & 0 \end{array}\right]$$

The Onsager operator K(q) is in the following form:

(137) $$\begin{array}{l} K\left(\rho ,z\right)=\left(\begin{array}{cc} 0 & 0\\ 0 & K(z) \end{array}\right)+\sum_{i=1}^{M}\left(\begin{array}{cc} \Theta_{C_{m}}^{Q_{m}} & -\Theta_{C_{m}}^{Q_{m}}B_{m}(z)\\ -B_{m}^{*}(z)\Theta_{C_{m}}^{Q_{m}} & B_{m}^{*}(z)\Theta_{C_{m}}^{Q_{m}}B_{m}(z) \end{array}\right)\\ \mathrm{with}\;B_{m}(z)DE(z)=H\;,\;\Theta_{C_{m}}^{Q_{m}}B_{m}(z)=\left[Q_{m}^{*},C_{m}\left[Q_{m},B_{m}(z)\right]\right] \end{array}$$

The dissipation operator is defined in terms of the dissipation potential, which is quadratic in the driving forces:

(138) $$DS\left(\rho ,z\right)=\left(\begin{array}{c} D_{\rho }S(\rho )\\ D_{z}S\left(\rho ,z\right) \end{array}\right)=\left(\begin{array}{c} -k_{B}\log\rho \\ D_{z}S(z) \end{array}\right)\;\mathrm{and}\;DE\left(\rho ,z\right)=\left(\begin{array}{c} H\\ D_{z}E\left(z\right) \end{array}\right)$$

The above assumptions guarantee a GENERIC system with the evolution equations given by the coupled system:

(139) $$\left\{\begin{array}{l} \frac{\partial \rho }{\partial t}=\frac{i}{\hbar }\left[\rho ,H\right]-\sum_{m=1}^{M}\Theta_{C_{m}(\rho ,z)}^{Q_{m}(z)}\left(k_{B}\log\rho +B_{m}(z)DS(z)\right)\\ \frac{\partial z}{\partial t}=K(z)DS(z)+\sum_{m=1}^{M}B_{m}^{*}(z)\Theta_{C_{m}(\rho ,z)}^{Q_{m}(z)}\left(k_{B}\log\rho +B_{m}(z)DS(z)\right) \end{array}\right.$$

Öttinger and Mielke argued that the dissipative superoperators $C_{m}$ should be given by the canonical correlation operator ${ℵ}_{\rho }$ (symmetric and positive semidefinite), which associates with the density matrix:

(140) $$\begin{array}{l} {ℵ}_{\rho }A=\int_{0}^{1}\rho^{s}A\rho^{1-s}ds\\ \mathrm{where}\;{ℵ}_{\rho }\mathrm{induces}\;\mathrm{the}\;\mathrm{scalar}\;\mathrm{product}\;{\langle A,B\rangle }_{\rho }=\langle {ℵ}_{\rho }A,B\rangle .\; \end{array}$$

The operator ${ℵ}_{\rho }$ is invertible.

(141) $${℘}_{\rho }A={ℵ}_{\rho }^{-1}A=\int_{0}^{\infty }{\left(\rho +sI\right)}^{-1}A{\left(\rho +sI\right)}^{-1}ds$$

The operator ${℘}_{\rho }A$ defines the Bogoliubov–Kubo–Mori metric on the set of density matrices.

There exists a connection to the von Neumann entropy because ${℘}_{\rho }A$ can be identified by its Hessian up to the factor

(142) $$-k_{B}:\langle A,D^{2}S(\rho )\rangle =-k_{B}\langle A,{℘}_{\rho }B\rangle$$

Kubo proved the following identity:

(143) $$\langle {ℵ}_{\rho }A,\log\rho \rangle =\langle A,\rho \rangle ={ℵ}_{\rho }\left[A,\log\rho \right]$$

that has been reused by Mielke to establish the relation between the von Neumann entropy and the canonical correlation operator.

(144) $$\begin{array}{l} \left\{\begin{array}{l} \frac{\partial \rho }{\partial t}=\frac{i}{\hbar }\left[\rho ,H\right]-\sum_{m=1}^{M}\left(\left[Q_{m},k_{B}\left[Q_{m},\rho \right]+{ℵ}_{\rho }\left(Q_{m},G_{m}(z)\right)\right]\right)\\ \frac{\partial z}{\partial t}=K(z)DS(z)+\sum_{m=1}^{M}B_{m}^{*}(z)\left[Q_{m},k_{B}\left[Q_{m},\rho \right]+{ℵ}_{\rho }\left(Q_{m},G_{m}(z)\right)\right] \end{array}\right.\\ \mathrm{with}\;G_{m}(z)=B_{m}(z)DS(z) \end{array}$$

We find the analogy with the classical Lindblad equation:

(145) $$\frac{\partial \rho }{\partial t}=\frac{i}{\hbar }\left[\rho ,H\right]-k_{B}\sum_{m=1}^{M}\left[Q_{m},\left[Q_{m},\rho \right]\right]$$

## 11. Synthesis

**Figure 5:**
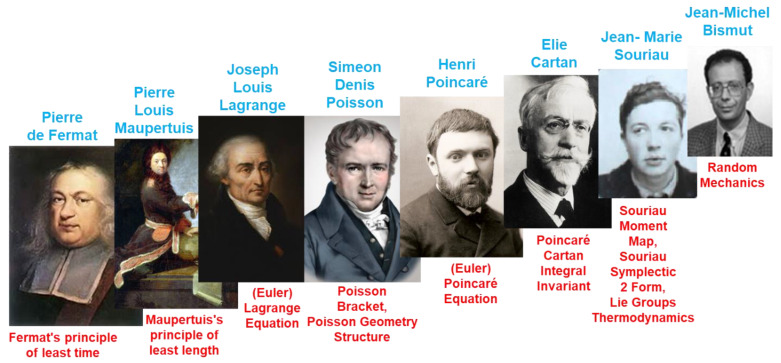
Main achievements in calculus of variations theory.

In this paper, we present a new Souriau Lie group thermodynamics as a symplectic foliation model of heat and information geometry. With the discovery of the “moment map,” Jean-Marie Souriau made a significant contribution to the calculus of variations, as illustrated in Figure 5, by introducing symplectic structures and geometrizing Noether’s theorem. Jean-Marie Souriau is regarded as a pioneer in the geometrization of the calculus of variations based on Lie groups and symmetries. He suggested a covariant model for thermodynamics and provided new geometric definitions of heat and Planck temperature from an ontological perspective.

Based on a symplectic model for statistical mechanics known as “Lie group thermodynamics,” proposed by Jean-Marie Souriau, we have developed the foliation geometry of thermodynamics. Entropy is an invariant Casimir function in coadjoint representation. Symplectic leaves, level sets of entropy, describe non-dissipative dynamics. On the other hand, symplectic leaves are described transversely for dissipative dynamics. The Koszul–Fisher metric extended by Jean-Marie Souriau on symplectic manifolds (as coadjoint orbit) with a moment map is the metric that is taken into consideration at this point. We could assume that transverse structures are related to Riemannian foliation given by the Koszul–Fisher metric.

In this model, geometric heat is a component of the dual Lie algebra, and geometric (Planck) temperature is a component of the Lie algebra of the group operating on the system. We have the characteristics that the Gibbs density is covariant and that the entropy and Koszul–Fisher–Souriau metric are invariants under the action of the group. Souriau addressed the general case of non-null cohomology in which a symplectic cocycle known as the Souriau cocycle emerges and the coadjoint operator is not equivariant (but “affine” equivariant).

The “transverse Poisson structure” connected to the Slodowy slice and Sabourin transverse structure describes the geometry of dissipative dynamics. Onsager–Casimir relations and the metriplectic and GENERIC models are addressed by Baptiste Coquinot. The Coquinot dissipative bracket is connected to the Onsager tensor, while the Poisson bracket represents non-dissipative dynamics (I have underlined also a link of the Onsager tensor with the Koszul–Fisher–Souriau tensor).

Because we just need to understand the Lie group acting on the thermodynamic system, this model is entirely intrinsic. We use it to describe dissipative quantum systems with a relation to the Dirac bracket in the Lindblad equation.

**Figure 6:**
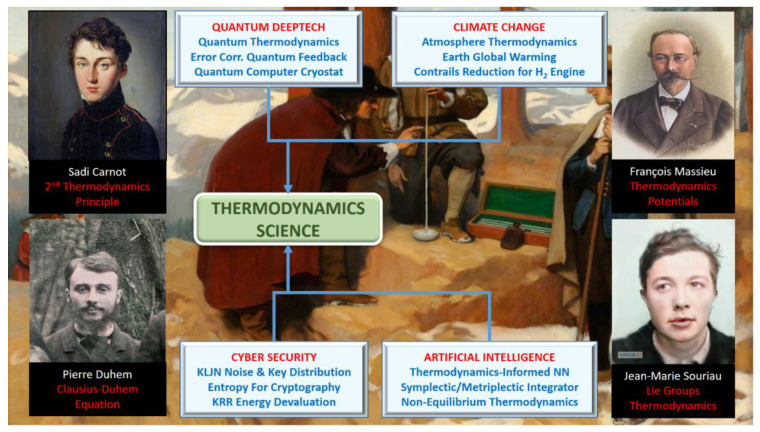
Geometric Thermodynamics Science for Quantum Deep Tech, Climate Change, Cyber Security and Artificial Intelligence.

These brand-new symplectic thermodynamic structures provide the way for new exploration in four crucial application domains, as illustrated in Figure 6:

- **Quantum deep tech**: quantum thermodynamics, error correction by quantum feedback on Lindblad equation;
- **Climate change**: atmosphere thermodynamics, Earth global warming studies, contrail reduction for H_2_ engine;
- **Cyber security**: KLJN noise and key distribution, entropy for cryptography, KRR energy devaluation;
- **Artificial intelligence**: thermodynamics-informed neural networks, symplectic and metriplectic integrator, non-equilibrium thermodynamics based on four pillars of contributions from Sadi Carnot (second principle of thermodynamics), François Massieu (thermodynamics potentials), Pierre Duhem (Clausius–Duhem equation) and Jean-Marie Souriau (Lie group thermodynamics).

## Data Availability

Not applicable.
